# The Composite Antiadhesion Barrier Facilitated Fibroblast Autophagy Activation for Tendon Repair

**DOI:** 10.1002/advs.202506204

**Published:** 2025-09-11

**Authors:** Zhenyu Sun, Jingwen Liu, Liang Chen, Zhen Wang, Fei Wang, Shen Liu

**Affiliations:** ^1^ Department of Orthopedics Shanghai Sixth People's Hospital Affiliated to Shanghai Jiao Tong University School of Medicine Shanghai 200233 P. R. China; ^2^ School of Nursing Shanghai Jiao Tong University No. 227 South Chongqing Road Shanghai 200025 P. R. China; ^3^ Department of Orthopedics Shanghai Key Laboratory for Prevention and Treatment of Bone and Joint Diseases Shanghai Institute of Traumatology and Orthopedics Ruijin Hospital Shanghai Jiao Tong University School of Medicine 197 Ruijin 2nd Road Shanghai 200025 P. R. China

**Keywords:** autophagy, IL‐37, on‐demand delivery, tendon adhesion, tendon repair, unidirectional delivery

## Abstract

While dysregulated autophagy is implicated in fibrotic processes in various organs, its specific role in peritendinous fibrosis and tendon adhesion formation remains to be elucidated. This study hypothesizes that autophagy exerts a protective effect, inhibiting adhesion formation following tendon injury. Furthermore, it is proposed that interleukin‐37 (IL‐37) can be a potential therapeutic target for preventing tendon adhesions through autophagy activation. Therefore, an innovative three‐layer composite antiadhesion barrier (pDNA@E–H–E′) equipped with on‐demand and unidirectional delivery strategy of bioactive plasmid DNA (pDNA) for IL‐37 overexpression is designed. The novel finding of the fibroblast's autophagy activity as a protective factor in tendon adhesion highlights the encapsulation of IL‐37‐encoding pDNA nanocomposites in barrier for reaching supreme antiadhesion efficacy. Moreover, the reactive‐oxygen‐species‐responsive and releasing‐direction‐guided pDNA@E–H–E′ membranes afford wonderful inhibition of fibroblast proliferation, fibroblast‐to‐myofibroblast differentiation, and collagen synthesis by enhancing autophagy. Further in a rat Achilles tendon adhesion model, pDNA@E–H–E′ membranes also significantly suppress peritendinous adhesion formation on the repaired sites and promote the scarless repair of Achilles tendon with optimum efficiency. In all, this study provides a promising approach for preventing tendon adhesion by employing a composite barrier with on‐demand and unidirectional delivery strategy.

## Graphical abstract

Synthesis of an innovative three‐layer composite antiadhesion barrier (plasmid DNA@E–H–E′) is schematically illustrated, highlighting its reactive‐oxygen‐species‐responsive, unidirectional interleukin‐37 delivery to enhance fibroblast autophagy, thereby effectively preventing tendon adhesion and promoting scarless tendon repair.

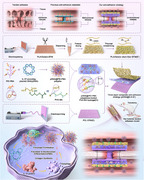

## 1 Introduction

Tendon adhesion, as one of the most common complications after tendon injury or surgery, tends to result in tendon gliding dysfunction and even extremity stiffness.^[^ ^1^ ^]^ Surgical intervention is often required to release the adhesive tissues, whereas the surgery would trigger adhesion recurrence, causing a vicious cycle of “adhesion, release, and readhesion.”^[^ ^2^ ^]^ Adhesion tissue formation between tendon and the surrounding tissues is considered a pathological fibrotic healing process, which involves inflammatory reactions, proliferation and activation of myofibroblast, and excessive collagen deposition.^[^ ^3^ ^]^

Autophagy is a highly conserved biological process, which involves phagocytizing structurally abnormal proteins and impaired or senescent organelles by double‐membrane autophagosomes and then fusing with lysosomes to form autophagolysosomes, in which the degradation is ultimately completed. Thus, autophagy provides an adaptive response to the situation of starvation, hypoxia and oxidative stress, pathogen infection, etc, thereby playing a critical role in achieving the organelle renewal and maintaining cell metabolism and homeostasis.^[^ ^4^, ^5^, ^6^, ^7^ ^]^ However, previous studies have indicated that autophagy dysfunction is involved in the pathogenesis of various diseases, such as neurodegeneration,^[^ ^8^ ^]^ cancer,^[^ ^9^ ^]^ diabetes,^[^ ^10^ ^]^ and cardiovascular disease.^[^ ^11^ ^]^ Existing evidences have also demonstrated that depressed autophagy is related to the development of pathological fibrosis of various tissues, such as heart,^[^ ^12^ ^]^ lung,^[^ ^13^ ^]^ and kidney.^[^ ^14^ ^]^ Moreover, the enhancement of autophagic activities brings about the decline of fibroblast proliferation, fibroblast‐to‐myofibroblast differentiation (FMD), and collagen synthesis, thereby ameliorating pathological fibrosis.^[^ ^15^, ^16^ ^]^ However, whether autophagy has a inhibitory role in peritendinous fibrosis still remains unclear. We suppose that autophagy activity may act as a prospective target for the intervention of tendon adhesion.

As a newly discovered member of the interleukin‐1 (IL‐1) family, interleukin‐37 (IL‐37) emerges as an anti‐inflammatory cytokine that suppresses innate inflammation and the adaptive immune responses, in contrast to most members of IL‐1 family, which function as proinflammatory mediators.^[^ ^17^ ^]^ It has been reported that administration of IL‐37 performed a beneficial role in preventing pulmonary inflammation and fibrosis induced by bleomycin (BLM), probably through attenuating inflammatory cells infiltration and decreasing the generation of pro‐inflammatory mediators, such as tumor necrosis factor‐α (TNF‐α) and interleukin‐6 (IL‐6), whereas increasing the production of anti‐fibroproliferative mediators, such as interferon‐γ.^[^ ^18^ ^]^ Mountford et al. recently reported that human IL‐37‐overexpressing transgenic mice are protected against CCl_4_‐induced liver fibrosis by functional interaction of IL‐37 with profibrogenic TGF‐β–Smad3 signaling cascade.^[^ ^19^ ^]^ More importantly, accumulating evidence has indicated that modulation of autophagy by IL‐37 sheds light on a novel perspective for pathogenesis and treatment of various diseases, such as tumor,^[^ ^20^ ^]^ inflammation,^[^ ^21^ ^]^ ischemia reperfusion injury,^[^ ^22^ ^]^ bone metabolism,^[^ ^23^ ^]^ etc. A recent study demonstrated that IL‐37 exerted tumor‐suppressive abilities in hepatocellular carcinoma (HCC) on account that IL‐37 positively regulated autophagy and inhibited the PI3K/AKT/mTOR signaling pathway in HCC cells.^[^ ^20^ ^]^ It was previously documented that the activation of autophagy functioned as an essential mechanism in enhanced osteogenic and odontogenic differentiation of dental pulp stem cells induced by IL‐37.^[^ ^23^ ^]^ Recently Kim et al. reported that a substantial decrease in IL‐37 levels was observed in the pulmonary tissues of patients with idiopathic pulmonary fibrosis (IPF) and BLM‐induced experimental lung fibrosis mice. Treatment with IL‐37 alleviated BLM‐mediated lung inflammation and fibrosis via the enhancement of autophagy and inhibition of TGF‐β1 receptor signaling in IPF fibroblasts, and administration of autophagy inhibitor 3‐methyladenine (3‐MA) could reverse this effect.^[^ ^24^ ^]^ Surprisingly, there have been no reports with respect to the correlation between IL‐37 and tendon adhesion so far, and the functional role and underlying mechanisms of IL‐37 in tendon adhesion remain unknown. Collectively, given the antifibrotic effects and enhanced autophagy activities of IL‐37, we hypothesized in this study that IL‐37 could be a promising therapeutic strategy for the prevention of tendon adhesion after tendon injury.

However, broken immune system homeostasis and undesired systemic toxicity make direct administration of the bioactive cytokines, such as IL‐37, an unfavorable choice owing to their immunogenicity, short circulating half‐life, and rapid proteolytic degradation in vivo, which demands repeated administration at a high price to achieve therapeutic effective concentrations.^[^ ^25^ ^]^ Gene therapy based on IL‐37‐encoding plasmid DNA (pDNA) emerges as a promising approach for producing long‐acting effects with optimal safety profile. Gene therapy is a technology that transfers and inserts functional and therapeutic genes using various carriers into target cells, thereby achieving long‐term and stable expression of target genes rather than repetitive administration of the recombinant proteins.^[^ ^26^ ^]^ Polyethyleneimine (PEI), considered as a favorable nonviral vector for gene transfection, can be further modified to improve the transfection efficiency due to abundant amino groups.^[^ ^27^ ^]^ Herein, we developed phenylboronic‐acid (PBA)‐modified PEI system (PEI─PBA) encapsulating IL‐37‐encoding plasmid DNA (pDNA@PEI─PBA) that effectively mediated the delivery of pDNA into cells and sustained expression of IL‐37.

**Scheme 1:**
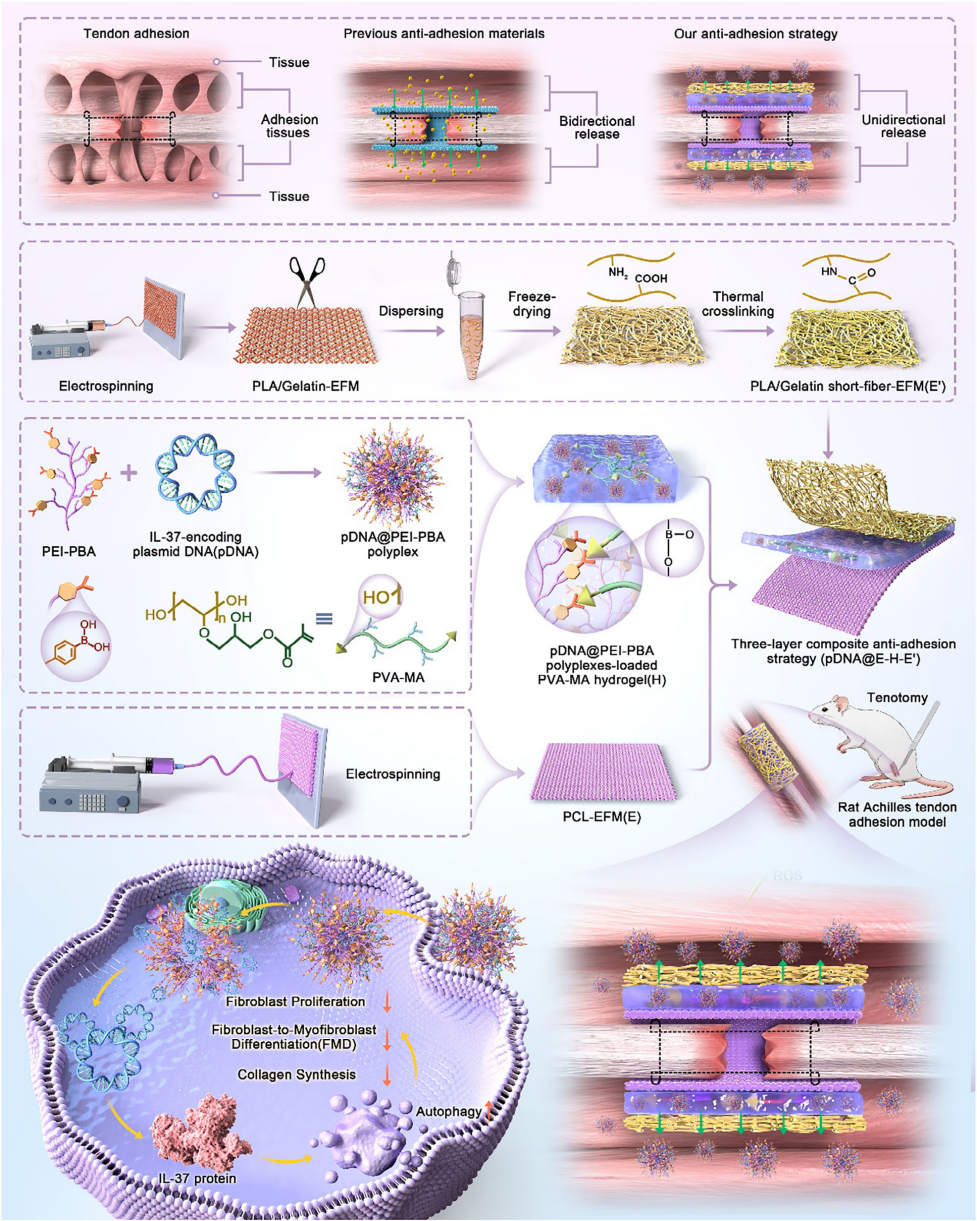
Overview of the proposed innovative antiadhesion strategy for tendon repair. Antiadhesion barrier materials are excellent strategies for preventing the formation of adhesion tissues, secondary to tendon repair operation or tendon injury. However, uncontrolled spatiotemporal release behaviors of previous antiadhesion materials design resulted in transient effect and unnecessary spread of therapeutic agents, thereby disrupting tendon intrinsic biological homeostasis and minimizing the therapeutic effects. In this study, an innovative three‐layer composite antiadhesion barrier (pDNA@E–H–E′) equipped with the ability of on‐demand and unidirectional delivery of bioactive plasmid DNA (pDNA) for IL‐37 overexpression was designed. First, a ROS‐responsive delivery mechanism (H) was introduced to the barrier by borate ester bond linkage through reactions between PVA─MA hydrogel and pDNA@PEI─PBA polyplexes. Then, the unidirectional release behavior could be informed by the porosity distinction in multilayer structure, manifested as the inner layer (E) of PCL‐EFM with lower porosity and the outer layer (Eʹ) of PLA/gelatin short‐fiber EFM with higher porosity. Moreover, our novel finding of the fibroblast's autophagy activity as a protective factor in tendon adhesion highlighted the encapsulation of IL‐37‐encoding pDNA nanocomposites in barrier for reaching supreme antiadhesion efficacy. Therefore, the ROS‐responsive and releasing‐direction‐guided pDNA@E–H–E′ membranes afforded wonderful inhibition of fibroblast proliferation, FMD, and collagen synthesis by enhancing autophagy. Further in a rat Achilles tendon adhesion model, pDNA@E–H–E′ also significantly suppressed tendon adhesion formation on the repaired sites and promoted the scarless repair of Achilles tendon with optimum efficiency.

As for the conventional antiadhesion strategies to apply drug‐loaded electrospun fibers or hydrogels, uncontrolled drug release performances become a daunting challenge due to the decline of actual therapeutic efficacy and disturbance of tendon inherent biological homeostasis. Therefore, drug delivery systems in response to peritendinous adhesion microenvironment emerge as a promising strategy to deliver therapeutic agents in peritendinous areas when specific pathological stimuli exist, thereby maximizing therapeutic efficiency and minimizing side effects.^[^ ^28^ ^]^ Specifically, generation of reactive oxygen species (ROS) probably works as a dominating trigger event in peritendinous adhesion microenvironment on account that overproduction of ROS is closely associated with pathogenesis of various diseases, such as inflammation, fibrosis, aging, and neurodegeneration.^[^ ^29^, ^30^, ^31^ ^]^ It has been reported that ROS increased in the tissues around the ruptured tendon at an early stage of tendon injury. The excessive production of ROS induced by hypoxia following tissue injuries results in the generation of oxidative stress (OS), which initiates the inflammation. The enhanced inflammation in turn exacerbates OS, ultimately leading to the adhesion formation.^[^ ^32^ ^]^ Thus, in this study, intelligent drug delivery system based on the boronic ester covalent bond between methacrylate grafted poly(vinyl alcohol) (PVA─MA) hydrogel and pDNA@PEI─PBA polyplexes was prepared for ROS‐responsive delivery of pDNA@PEI─PBA polyplexes into peritendinous adhesion areas. Moreover, an ideal antiadhesion system is supposed to be equipped with unidirectional delivery properties for fear of the interference with tendon healing. In our previous works, diverse electrospun fibrous membranes (EFMs) have been fabricated as physical barriers showing excellent antiadhesion effects partially due to the tightly packed structures for preventing unexpected molecule/cell diffusion.^[^ ^33^ ^]^ Interestingly, our team also innovatively developed short fiber‐based electrospun fibrous membranes/scaffolds with larger pore size and higher porosity, achieving more facile exchange of molecules between the inside and outside.^[^ ^34^ ^]^ Herein, three‐layer composite antiadhesion platform (pDNA@E–H–E′) composed of polycaprolactone (PCL)‐EFM with lower porosity as the inner layer (E), pDNA@PEI─PBA polyplexes‐loaded PVA─MA hydrogel as the middle layer (H), and poly lactic acid (PLA)/gelatin short‐fiber EFM with higher porosity as the outer layer (E′) was for the first time introduced for on‐demand and unidirectional delivery of IL‐37 (**Scheme** **1**).

## 2 Results and Discussion

### 2.1 Inflammation, Oxidative Stress, Autophagy Activity, and IL‐37 Levels in the Development of Tendon Adhesion

**Figure 1:**
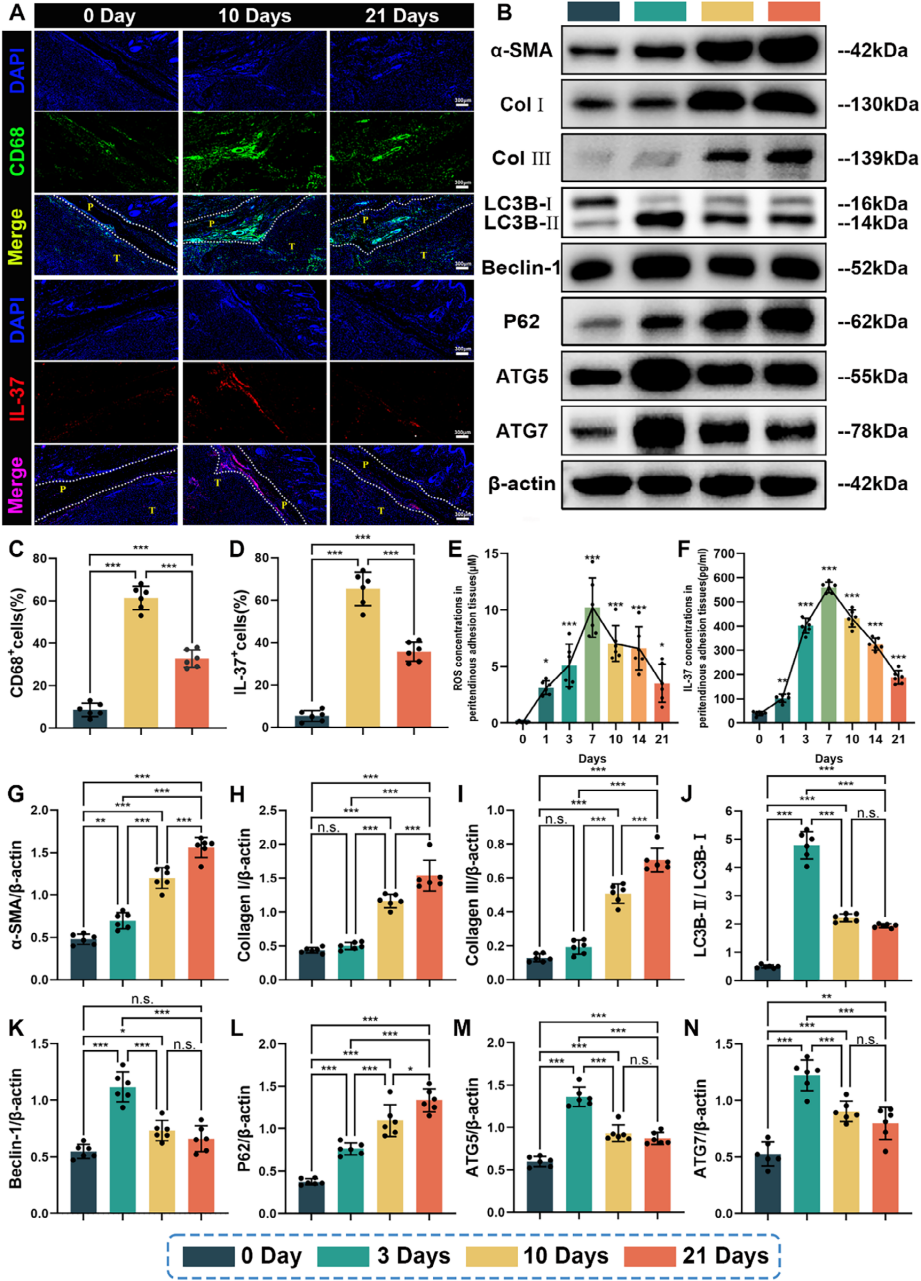
IL‐37 contents, ROS concentrations, autophagy, and fibrosis levels during the course of rat Achilles adhesion model. A) Representative fluorescence staining images of CD68^+^ (green) and IL‐37^+^ (red) cells in peritendinous adhesion tissues at 0 day, 10 days, and 21 days postoperation. Percentages of the C) CD68^+^ cells and D) IL‐37^+^ cells in peritendinous adhesion tissues. Concentrations of E) ROS and F) IL‐37 in peritendinous adhesion tissues at various time points (0, 1, 3, 7, 10, 14, and 21 days) during the progression of the rat Achilles adhesion model. (B) The protein expressions of key markers associated with autophagy (LC3B, Beclin‐1, P62, ATG5, and ATG7) and fibrosis (Collagen I, Collagen III, and α‐SMA) in peritendinous adhesion tissues at 0, 3, 10, and 21 days following surgery were determined by western blotting. G) α‐SMA, H) Collagen I, I) Collagen III, J) LC3B, K) Beclin‐1, L) P62, M) ATG5, and N) ATG7 expressions were normalized to β‐actin (mean ± SD, *p < 0.05, **p < 0.01, ***p < 0.001, *n* = 6).

As tissue hypoxia and inflammation following tissue damage can disturb redox equilibrium, thereby initiating fibrotic responses, correlation among inflammation, oxidative stress, and tendon adhesion formation was investigated in this study. It can be distinctly observed that there were abundant CD68^+^ cells (CD68 is predominantly expressed in macrophages) infiltration in the peritendinous adhesion tissues in the group of 10 days postoperation, compared with the control group (0 day postoperation). At 21 days postoperation, the proportion of the CD68^+^ cells in the peritendinous adhesion tissues decreased (Figure **1**A,C). It has been reported that the activation of platelet‐derived cytokine signaling caused enhanced vascular permeability and the attraction of circulating inflammatory cells to the damage sites of tendon immediately after acute injury.^[^ ^35^, ^36^ ^]^ During the inflammatory phase of tendon healing, macrophages have been shown to predominate over other cell types at the damage sites, especially in the peritendinous tissues.^[^ ^35^, ^37^ ^]^ In addition, it is obviously seen from our results that ROS concentrations in the peritendinous adhesion tissues rose up 24 h later, peaked at 7 days, and then decreased gradually over time, indicating a close correlation between inflammation and oxidative stress (Figure 1E). Inflammation following tendon injury can promote the excessive production of ROS and aggravate the oxidative stress.^[^ ^38^ ^]^ Meanwhile, oxidative stress will, in turn, exacerbate the inflammatory response by increasing vascular permeability and the release of inflammatory factors, such as TNF‐α, IL‐1, and IL‐6.^[^ ^39^ ^]^ It has been demonstrated that ROS are essential mediators of fibrogenesis and might be responsible for initiating the process of fibrosis, finally resulting in formation of unnecessary tendon adhesion.^[^ ^32^, ^40^ ^]^ Previous studies have revealed that antagonizing posttraumatic oxidative stress by Trolox (a Vitamin‐E analog) and Vitamin‐C, both of which are strong antioxidants, could reduce the extent of tendon adhesions by modulating oxidative stress and redox.^[^ ^41^, ^42^ ^]^ Therefore, antioxidation strategies and drug delivery strategies in response to the local oxidative stress are both in urgent need for preventing peritendinous fibrosis as soon as the tendon injury occurs.

Then, to investigate autophagy levels in the progress of tendon adhesion, we harvested the peritendinous tissues from rats subjected to Achilles tendon injury and evaluated LC3B, Beclin‐1, P62, ATG5, and ATG7 levels at 0, 3, 10, and 21 days, as vital autophagy markers. Western blotting analyses indicated that the protein levels of LC3B, Beclin‐1, P62, ATG5, and ATG7 were all relatively low in peritendinous tissues from the control group (0 day postoperation), suggesting low activity of autophagy in normal peritendinous tissues (Figure 1B,J–N). After tendon injury, the expressions of LC3B, Beclin‐1, ATG5, and ATG7 temporarily increased during the initial stages and significantly decreased at 10 and 21 days (Figure 1B,J,K,M,N). However, the protein levels of p62 gradually increased after tendon injury and peaked at 21 days, showing reduced p62 degradation (Figure 1B,L). Meanwhile, we also evaluated protein levels of α‐smooth muscle actin (α‐SMA), Col I, and Col III, as vital fibrotic markers. Western blotting analyses showed that compared with the control group, the expressions of α‐SMA, Col I, and Col III all notably increased at 10 days after injury, and peaked at 21 days after injury, indicating that fibroblast‐to‐myofibroblast differentiation and collagen synthesis significantly increased since autophagy started to diminish (Figure 1B,G–I). These results highlighted the potential negative correlation between autophagy activity and injury‐induced peritendinous fibrosis.

Finally, the content variation of IL‐37 in peritendinous tissues in the development of peritendinous fibrosis was investigated in this study. As shown in Figure 1A,D, in comparison with the control group, obvious infiltration of IL‐37‐positive cells into the peritendinous adhesion tissues was observed from the group of 10 days postoperation. In addition, the group of 21 days postoperation showed a decrease in the infiltration of IL‐37‐positive cells (Figure 1A,D). Moreover, the enzyme‐linked immunosorbent assay (ELISA) results indicated that IL‐37 concentrations in the peritendinous tissues increased after tendon injury, peaked at 7 days, and then decreased gradually over time (Figure 1F). Previous studies have demonstrated that IL‐37 can regulate innate immune responses and inhibit the production of proinflammatory cytokines and chemokines through negative feedback regulation mechanism.^[^ ^17^, ^43^, ^44^ ^]^ Given the evidence above, during the initial inflammatory phase of tendon healing, the increased IL‐37 was involved in a negative feedback loop to manage the excessive inflammation. However, during the fibroblastic/proliferative phase, IL‐37 levels in peritendinous tissues correlated negatively with fibrotic changes after injury‐induced peritendinous fibrosis. IL‐37 could be a therapeutic target for the intervention of tendon adhesion.

### 2.2 Characterization and Transfection Efficiency of pDNA@PEI─PBA Polyplexes

**Figure 2:**
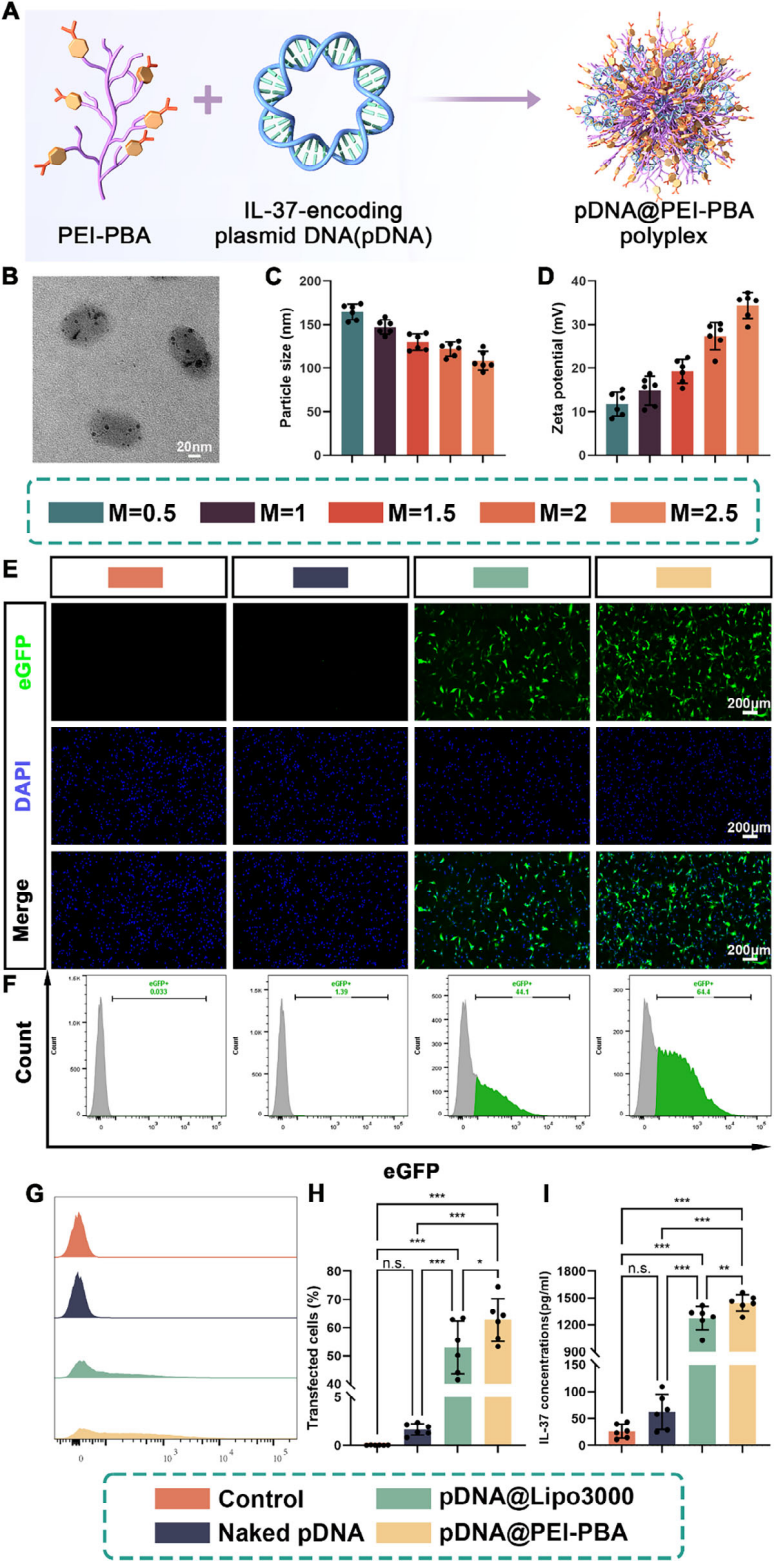
Synthesis and characterization of pDNA@PEI─PBA polyplex for IL‐37‐encoding plasmid DNA delivery. A) Schematic diagram of the combination of PEI─PBA and IL‐37‐encoding pDNA to form transfection polyplexes. B) Representative TEM image of pDNA@PEI─PBA polyplexes. The C) particle size distribution and D) zeta potentials of pDNA@PEI─PBA polyplexes at different mass ratios were measured using DLS. Transfection efficiency of rat fibroblast 208F cell lines subjected to different treatments was determined by E) fluorescence microscopy and (F, G) flow cytometric (FCM) assays. F–H) Transfection efficiency as the percentage of eGFP‐positive cells was determined by FCM. I) Quantification of the IL‐37 secretion after 5 days of transfection (mean ± SD, *p < 0.05, **p < 0.01, ***p < 0.001, *n* = 6).

In order to achieve the high transfection efficacy of IL‐37 plasmid, previously reported PEI─PBA nonviral gene vector was used and positively charged PEI─PBA polymer could bind to anionic plasmid DNA through electrostatic interaction to form stabilized nanoscaled polyplexes (pDNA@PEI─PBA).^[^ ^45^, ^46^ ^]^ First, the morphology of pDNA@PEI─PBA polyplexes was observed by transmission electron microscopy (TEM), showing typical near‐spherical structure (Figure **2**B). Given that gene transfection efficiency is directly influenced by the particle size and surface potential of cationic delivery vehicles, we proceeded to further characterize the pDNA@PEI─PBA polyplexes. It was found by dynamic light scattering (DLS) analyzer that as the mass ratios [*M* = *M*(PEI─PBA)/*M*(pDNA)] of pDNA@PEI─PBA polyplexes increased, their surface potentials significantly rose from 11.78 ± 2.73 to 34.36 ± 2.96 mV (Figure 2D). In addition, the particle sizes of the pDNA@PEI─PBA polyplexes demonstrated a significant decrease from 164.45 ± 8.77 to 108.39 ± 10.77 nm with an increase in the mass ratios of the polyplexes, likely due to enhanced positive charge compressing negatively charged pDNA and forming more compact structures (Figure 2C). These favorable biophysical properties of the transfected nanoparticles contribute to efficient cellular uptake and nucleic acid delivery.^[^ ^47^ ^]^ To screen an appropriate mass ratio of pDNA@PEI─PBA polyplexes for gene delivery, we determined the transfection efficiency for rat fibroblast 208F cell lines using fluorescence microscopy and flow cytometry (FCM). The results showed that the transfection efficiency was the highest at *M* = 2 with lower cytotoxicity, which was chosen as the optimal condition to conduct the subsequent studies (Figures S5–S7, Supporting Information). In order to further investigate the transfection efficacy of pDNA@PEI─PBA polyplexes, fluorescence imaging and flow cytometry were performed and lipofectamine 3000 (Lipo 3000), as the commercial transfection reagent, was introduced as positive control.^[^ ^48^ ^]^ As shown in Figure 2E, compared with pDNA alone and pDNA@Lipo 3000, pDNA@PEI─PBA group exhibited the highest fluorescence intensity of enhanced green fluorescent protein (eGFP) in 208F cells. Meanwhile, the results of flow cytometry and percentage of eGFP‐positive cells determined by FCM were in accordance with fluorescence imaging results (Figure 2F–H). In addition, the transfection efficacy of pDNA@PEI─PBA polyplexes on 208F cells was also tested by measuring the secretion of IL‐37 using ELISA. The results indicated that the maximum secretion of IL‐37 was obtained in the pDNA@PEI─PBA group after transfection for 5 days (1444.26 ± 92.27 pg mL^−1^), which was superior to that in pDNA@Lipo 3000‐treated group (1274.92 ± 132.52 pg mL^−1^) (Figure 2I). The results were consistent with aforementioned results.

### 2.3 pDNA@PEI─PBA Polyplexes Diminished TGF‐β1‐Induced Fibroblast Proliferation, FMD, and Collagen Synthesis in Rat Fibroblast 208F Cell Lines by Enhancing Autophagy

Excessive fibroblast proliferation, myofibroblast activation, and resultant extracellular matrix (ECM) overproduction have been identified as key pathological features of tendon adhesion.^[^ ^49^ ^]^ The pathogenesis of tendon adhesion involves multiple regulatory mechanisms, among which TGF‐β1‐mediated signaling pathways have been identified as pivotal modulators.^[^ ^50^ ^]^ Compelling evidence identifies TGF‐β1 as the predominant regulator of myofibroblast phenotype, driving the transition of quiescent fibroblasts to α‐SMA‐positive (differentiated) myofibroblasts.^[^ ^51^ ^]^ Moreover, by disrupting the homeostatic balance of cell cycle progression, TGF‐β1 activation induces pathological hyperproliferation in affected cell populations.^[^ ^52^ ^]^ Fibroblasts serve as the principal effector cells mediating tendon adhesion pathogenesis, and therefore rat fibroblast 208F cell lines were treated as the cell model in this study.

**Figure 3:**
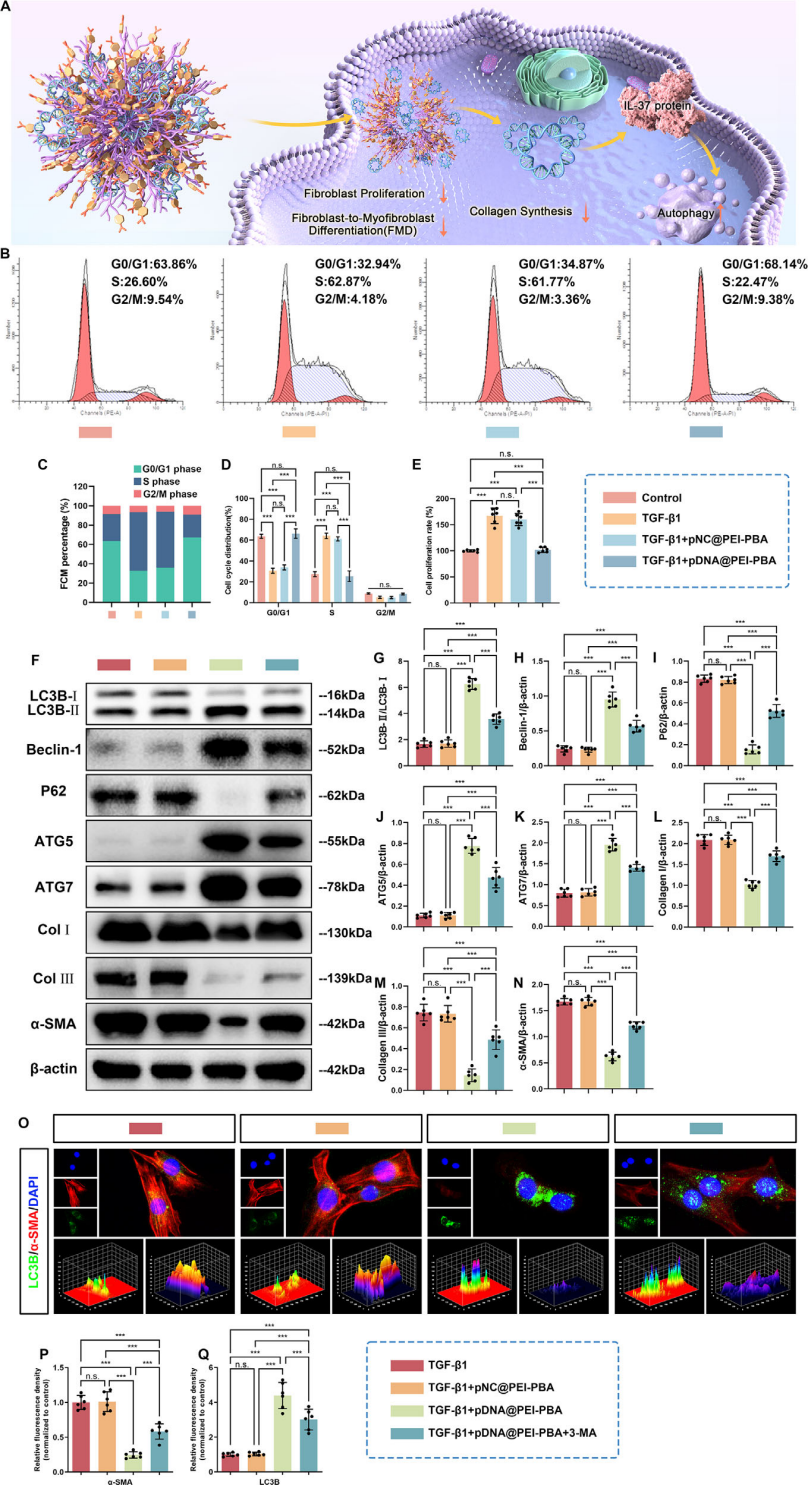
pDNA@PEI─PBA polyplexes diminished TGF‐β1‐induced fibroblast proliferation, FMD, and collagen synthesis in rat fibroblast 208F cell lines by enhancing autophagy. A) Schematic diagram of mechanisms of pDNA@PEI─PBA‐polyplexes‐mediated antiadhesion effects. B) Representative flow cytometry images and C,D) the corresponding quantitative analysis of cell cycle distribution following various treatment conditions. E) Cell proliferation analysis of 208F cells after different treatments by CCK‐8. F) The protein expressions of key markers associated with autophagy (LC3B, Beclin‐1, P62, ATG5, and ATG7) and fibrosis (Collagen I, Collagen III, and α‐SMA) in 208F cells after different treatments were determined by western blotting. G) LC3B, H) Beclin‐1, I) P62, J) ATG5, K) ATG7, L) Collagen I, M) Collagen III, and N) α‐SMA expressions were normalized to β‐actin. O) Representative immunofluorescence images showing costaining of LC3B (green) and α‐SMA (red) in 208F cells subjected to different treatments. Semiquantitative analysis of fluorescence intensity for P) α‐SMA and Q) LC3B (mean ± SD, *p < 0.05, **p < 0.01, ***p < 0.001, *n* = 6).

First, flow cytometric analysis revealed distinct cell cycle distribution in 208F cells after different treatments. As shown in Figure **3**B–D, compared with the control group, TGF‐β1 disrupted normal cell cycle kinetics by prolonging S and G2/M phase transition. Notably, addition of pDNA@PEI─PBA polyplexes to TGF‐β1‐treated 208F cells effectively reversed this dysregulation, achieving a significant increase in the proportion of cells arrested in G0/G1 phase, compared to both TGF‐β1 monotherapy and TGF‐β1 + pNC@PEI─PBA cotreatment groups (Figure 3B–D). Additionally, fibroblast proliferative viability was determined through cell counting kit‐8 (CCK‐8) assay. The results in Figure 3E revealed a marked increase in fibroblast viability following TGF‐β1 stimulation. However, this proproliferative effect was significantly attenuated by pDNA@PEI─PBA polyplexes cotreatment (Figure 3E). Collectively, these findings indicated that therapeutic targeting of cell cycle progression through pDNA@PEI─PBA polyplexes delivery significantly attenuated TGF‐β1‐mediated pathological fibroblast proliferation via sustained G0/G1 arrest, providing a novel strategy to disrupt TGF‐β1‐mediated fibrogenesis.

The effect of IL‐37 overexpression on autophagy level, FMD, and collagen synthesis in fibroblasts was investigated by detecting the expressions of autophagy‐associated proteins and fibrosis‐associated proteins in 208F cells after transfection with pDNA@PEI─PBA polyplexes. As demonstrated in Figure 3F–N, TGF‐β1 + pDNA@PEI─PBA treatment group exhibited substantially elevated expression levels of autophagy‐related markers LC3B, Beclin‐1, ATG5, and ATG7 compared to both TGF‐β1 monotherapy and TGF‐β1 + pNC@PEI─PBA treatment groups. Conversely, this therapeutic intervention showed a marked reduction in the expression of P62 and fibrotic markers α‐SMA, Col I, and Col III relative to the other two groups (Figure 3F–N). Notably, western blotting analysis revealed that pretreatment with 3‐MA, an autophagy inhibitor, effectively reversed these observed trends (Figure 3F–N). This reversal was evidenced by the inhibition of LC3B, Beclin‐1, ATG5, and ATG7 accumulation and a decrease in the degradation of P62. Importantly, 3‐MA also abrogated the inhibitory effects of pDNA@PEI─PBA polyplexes on TGF‐β1‐induced FMD and collagen synthesis (Figure 3F–N). Collectively, these results demonstrated that the overexpression of IL‐37 after transfection with pDNA@PEI─PBA polyplexes contributed to the enhancement of autophagy activities in fibroblasts and provided a protective effect against fibrogenesis through an autophagy‐dependent mechanism. In addition, the results of immunofluorescence double staining for LC3B (an autophagy marker) and α‐SMA (a myofibroblast marker) in 208F cells following various treatments confirmed the aforementioned conclusions (Figure 3O–Q). Figure 3O–Q illustrated that the expression level of LC3B in TGF‐β1 + pDNA@PEI─PBA treatment group was notably greater than those in both TGF‐β1 monotherapy and TGF‐β1 + pNC@PEI─PBA treatment groups, whereas the expression level of α‐SMA in TGF‐β1 + pDNA@PEI─PBA treatment group was significantly lower than those in the other two groups, indicating that IL‐37 overexpression after pDNA@PEI─PBA polyplexes transfection promoted autophagy in fibroblasts and conferred protection against fibrogenesis. Not surprisingly, pharmacological inhibition of autophagy with 3‐MA not only attenuated the upregulation of LC3B, but also effectively abrogated the suppressive effects of pDNA@PEI─PBA polyplexes on TGF‐β1‐mediated FMD (Figure 3O–Q). Our data strongly suggested that the observed antifibrotic effects were mechanistically dependent on autophagy activation.

### 2.4 Characterization of pDNA@E–H–E′ Composite Membranes

The three‐layer composite antiadhesion membrane (pDNA@E–H–E’) was equipped with advantages as follows: 1) PCL‐EFM with lower porosity could act as an effective physical barrier to prevent unexpected therapeutic agents diffusion toward injured tendon side, which was beneficial for avoiding impairing the tendon intrinsic healing; 2) PVA─MA hydrogel could efficiently load pDNA@PEI─PBA polyplexes via ROS degradable borate ester bond, and the ROS‐responsive delivery mechanism could realize on‐demand release of nanocomposites based on the changing pathological microenvironment, thereby improving both safety and efficiency of the delivery strategies; 3) PLA/gelatin short‐fiber EFM with higher porosity permitted the passage of pDNA@PEI─PBA polyplexes to peritendinous areas, stabilized the structure of inner hydrogel, and thus promoted therapeutic effects.

**Figure 4:**
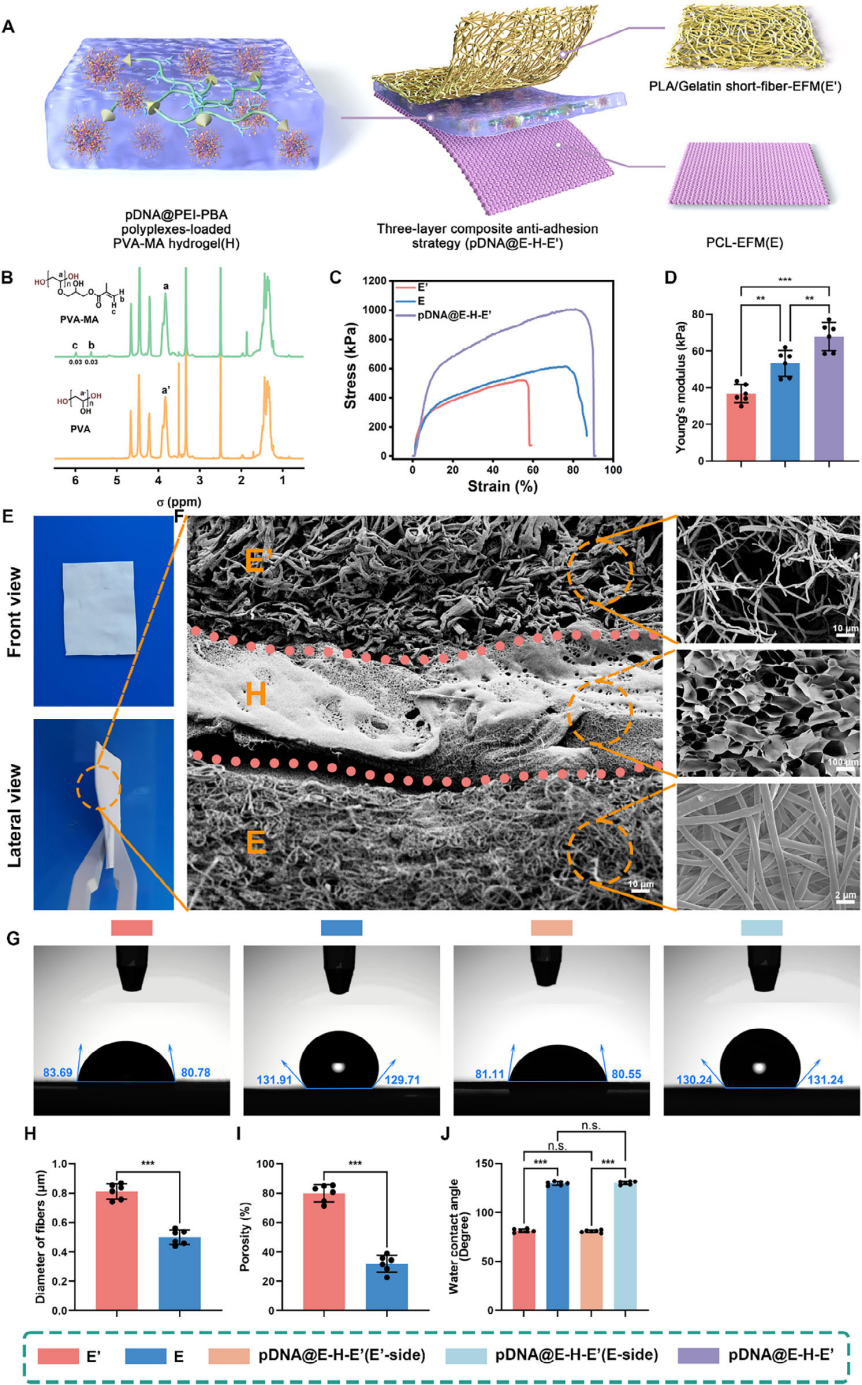
Constitution and characterization of the three‐layer composite antiadhesion membrane (pDNA@E–H–E′). A) Schematic illustration of the constitution of pDNA@E–H–E′. B) ^1^H NMR spectra of PVA─MA and the pristine PVA. C) Tensile mechanical properties of PLA/gelatin short‐fiber EFM (E′), PCL‐EFM (E), and pDNA@E–H–E′ as indicated by stress–strain curves. D) Statistical graphs of Young's modulus of three membranes. E) The front view and lateral view of pDNA@E–H–E′ composite antiadhesion membrane. F) Representative cross‐sectional SEM image of the three‐layer composite antiadhesion membrane (pDNA@E–H–E′). Representative SEM images of PLA/gelatin short‐fiber‐EFM (E′) layer, pDNA@PEI─PBA‐polyplexes‐loaded PVA─MA hydrogel (H) layer, and PCL‐EFM (E) layer. G) Representative water contact angle images of different membranes. Statistical analysis of H) diameter of fibers, I) porosity, J) water contact angles of different membranes (mean ± SD, *p < 0.05, **p < 0.01, ***p < 0.001, *n* = 6).

First, PVA─MA was initially characterized using ^1^H NMR (Figure **4**B). Compared to the spectrum of the pristine PVA, new peaks at 6.0 and 5.6 ppm were observed, corresponding to the characteristic double bonds of the MA group. This finding indicated that the methacrylate group was successfully grafted to the pendant hydroxyl groups of PVA. The degree of substitution of PVA─MA was determined by integrating the areas under the characteristic methacrylate peaks at 6.0 and 5.6 ppm, relative to the main chain peak of PVA at 3.8 ppm.

In order to wrap the disrupted tendon and achieve unidirectional release of nanocomposites toward peritendinous areas, pDNA@E–H–E′ composite antiadhesion membrane was fabricated by combing PCL‐EFM (E) as the inner layer, pDNA@PEI─PBA‐polyplexes‐loaded PVA─MA hydrogel (H) as the middle layer, and PLA/gelatin short‐fiber EFM (E′) as the outer layer, as revealed by the front view and lateral view (Figure 4E). The cross‐sectional scanning electron microscopy (SEM) images (Figure 4F) of pDNA@E–H–E′ membrane indicated that the composite membrane exhibited a three‐layer structure with clear boundaries and hydrogel layer was blended into nanofibers. The high dense fiber distribution with low porosity of PCL‐EFM layer restricted nanocomposites’ access to the tendon, whereas it permitted the passage of nutrients to facilitate tendon intrinsic healing.^[^ ^28^ ^]^ Meanwhile, the prominent difference in porosity between PCL‐EFM layer and PLA/gelatin short‐fiber‐EFM layer made it possible to unidirectionally delivery nanocomposites toward peritendinous areas (Figure 4H,I). To better characterize the surface wettability of the membranes, water contact angles were measured for PLA/gelatin short‐fiber EFM, PCL‐EFM, and both sides of the asymmetric pDNA@E–H–E′ composite membrane. The contact angles were determined as follows: PLA/gelatin short‐fiber EFM (81.34° ± 1.68°), PCL‐EFM (129.69° ± 1.8°), pDNA@E–H–E′ (E′‐side: 80.67° ± 1.28°; E‐side: 130.26° ± 1.58°), as shown in Figure 4G and quantified in Figure 4J. These results clearly demonstrate the asymmetric wettability of the composite membrane, with the E′‐side exhibiting hydrophilic characteristics and the E‐side being distinctly hydrophobic. From the stress–strain curves, we found that the tensile mechanical properties of pDNA@E–H–E′ composite membrane were reinforced compared to PLA/gelatin short‐fiber EFM and PCL‐EFM alone (Figure 4C,D). Figure 4D illustrated that the Young's modulus of pDNA@E–H–E′ (67.82 ± 7.77 kPa) was significantly higher than those of PLA/gelatin short‐fiber EFM (36.73 ± 5.02 kPa) and PCL‐EFM (53.22 ± 7.08 kPa).

### 2.5 In Vitro Unidirectional and ROS‐Responsive Release Properties of pDNA@E–H–E′

**Figure 5:**
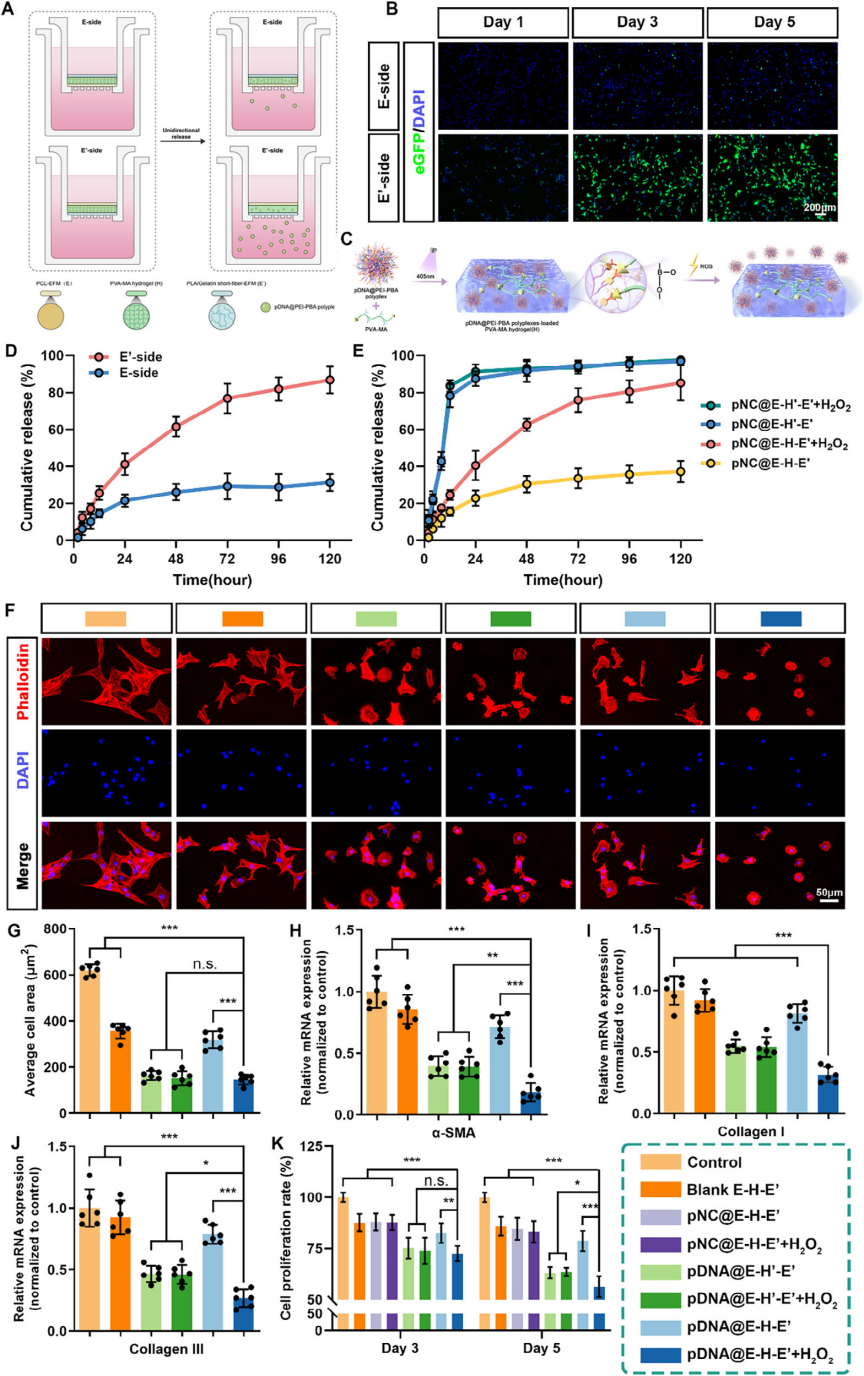
Unidirectional release and ROS‐responsive release properties and antiadhesion properties of pDNA@E–H–E′. A) Schematic illustration of polyplexes release and cell transfection from both two sides (Eʹ‐side and E‐side) of the pDNA@E–H–E′ by using Transwell plates. B) Fluorescence images of pDNA@PEI─PBA‐polyplexes‐transfected 208F cells for 1, 3, and 5 days. C) Schematic illustration of preparation of pDNA@PEI─PBA‐polyplexes‐loaded PVA─MA hydrogel, formation of borate ester bond linkage, and polyplexes release in response to ROS. D) Quantification of unidirectional release of pDNA@PEI─PBA polyplexes from the Eʹ‐side and E‐side of pDNA@E–H–E′ in H_2_O_2_ solutions. E) Quantification of nanocomposites release in response to ROS from pDNA@E–Hʹ–Eʹ and pDNA@E–H–E′ in the presence or absence of H_2_O_2_. F) Cell morphology and adhesion analysis of 208F cells cocultured with various membranes for 3 days, and G) the corresponding statistical analysis of average cell area. qRT‐PCR analysis of mRNA expressions of H) α‐SMA, I) Collagen I, and J) Collagen III in 208F cells. K) Proliferation ability of 208F cells cocultured with various membranes for 3 and 5 days was examined using CCK‐8 assay (mean ± SD, *p < 0.05, **p < 0.01, ***p < 0.001, *n* = 6).

Due to the asymmetrical structure with distinguishing porosities of pDNA@E–H–E′ composite antiadhesion membrane, it can be presumed that pDNA@PEI─PBA polyplexes release from pDNA@E–H–E′ was unidirectional. The unidirectional release assay was performed in Transwell plates, and the cumulative release curves of pDNA@PEI─PBA polyplexes from pDNA@E–H–E′ membranes in the presence of H_2_O_2_ were shown in Figure **5**D. Of note, the cumulative release level of pDNA was much higher when the PLA/gelatin short‐fiber‐EFM (E′) layer was at the bottom of the upper chamber compared with the PCL‐EFM (E) layer at the bottom of the upper chamber, indicating that in the pDNA@E–H–E′ composite antiadhesion membrane, the PCL‐EFM (E) layer with lower porosity as inner layer served as an effective physical barrier to restrain pDNA@PEI─PBA polyplexes, while the PLA/gelatin short‐fiber‐EFM (E′) layer with higher porosity as outer layer created an accessible avenue for unidirectional release of pDNA@PEI─PBA polyplexes to peritendinous side. Moreover, the results of fluorescence imaging for eGFP in pDNA@PEI─PBA‐polyplexes‐transfected 208F cells for 1, 3, and 5 days also confirmed the aforementioned conclusions (Figure 5B). Pronounced perinuclear green fluorescence intensification was documented specifically in transfected 208F cells on the Eʹ‐side on the third and fifth days, demonstrating that a substantial amount of pDNA@PEI─PBA polyplexes were released from the Eʹ‐side of pDNA@E–H–E′ and subsequently internalized by the cells. By contrast, cells on the E‐side showed no noticeable change in fluorescence intensity, suggesting limited release and uptake of the polyplexes (Figure 5B). Meanwhile, the results of flow cytometry and percentage of eGFP‐positive cells determined by FCM were in accordance with fluorescence imaging results (Figure S8, Supporting Information). In short, this polarized release pattern provides critical insights into the carrier system's unidirectional delivery mechanism.

The ROS‐responsive release behavior of nanocomposites from pDNA@E–H–E′ composite antiadhesion membrane was investigated in this study. The cumulative release curves of nanocomposites from pDNA@E–Hʹ–Eʹ and pDNA@E–H–E′ membranes in the presence or absence of H_2_O_2_ were shown in Figure 5E (Hʹ: non‐ROS‐responsive PVA─MA hydrogel loaded with pDNA@PEI polyplexes; H: ROS‐responsive PVA─MA hydrogel loaded with pDNA@PEI─PBA polyplexes). Biomedical materials based on borate ester bond linkage are highly sensitive to H_2_O_2_.^[^ ^53^, ^54^ ^]^ Not surprisingly, H_2_O_2_ notably promoted the release of pDNA@PEI─PBA polyplexes from pDNA@E–H–E′ and the cumulative release level of pDNA@PEI─PBA polyplexes from pDNA@E–H–E′ with H_2_O_2_ stimulation was more than 3 times than that without H_2_O_2_ stimulation (Figure 5E). These results indicated that pDNA@PEI─PBA polyplexes release from hydrogel triggered by H_2_O_2_, and pDNA@E–H–E′ exhibited on‐demand release in respond to elevated ROS microenvironment of peritendinous adhesion areas. In addition, these results demonstrated that pDNA@E–H–E′ did not exhibit burst release of pDNA@PEI─PBA polyplexes but rather achieve sustainable and stable release of pDNA@PEI─PBA polyplexes over the course of at least 5 days, at the end of which cumulative release was more than 80% (Figure 5E). However, in the presence or absence of H_2_O_2_, non‐ROS responsive pDNA@E–Hʹ–Eʹ membrane without borate ester bond linkage exhibited rapid burst release of nanocomposites during the first 12 h, followed by a release kinetic less than 30 h. Following a classic wound healing response, tendon healing process after tendon injury can be divided into three consecutive and overlapping stages: 1) inflammation stage (lasting days), 2) proliferation stage (weeks), and 3) remodeling stage (months to years); besides, the exact duration of each stage depends on tendon location, injury degree, therapeutic method, and other complications.^[^ ^55^ ^]^ Tendon adhesions were considered as an abnormal healing process characterized by initial inflammatory responses, subsequent myofibroblast proliferation and activation, and excessive ECM deposition.^[^ ^36^ ^]^ Hence, it could be inferred that pDNA@E–H–E′ composite antiadhesion membrane could achieve on‐demand, sustainable, and unidirectional delivery of IL‐37 into the target area, which matched the progression of tendon adhesion and was suitable for application in antiadhesion treatments.

### 2.6 Evaluation of Anticell Adhesion, Fibrosis‐Associated Gene Expressions, Cell Proliferation Inhibition of pDNA@E–H–E′ In Vitro

After 3 days of coculture, the cytoskeleton and nuclei of rat fibroblast 208F cell lines were dyed red and blue, respectively, and the adhesion of cells growing on the surfaces of various membranes was compared (Figure 5F,G). Figure 5F demonstrated that 208F cells in the control group exhibited well‐organized and highly extensible cytoskeletal arrangements. However, the pDNA@E–H–E′ + H_2_O_2_ group showed significant reductions in the average cell areas compared to control, Blank E–H–E′, and pDNA@E–H–E′ groups. No significant differences in average cell areas were observed among groups of pDNA@E–Hʹ–Eʹ, pDNA@E–Hʹ–Eʹ + H_2_O_2_ and pDNA@E–H–E′ + H_2_O_2_ (Figure 5F,G). In addition, the proliferation of 208F cells among all groups was also investigated after 3 and 5 days of coculture (Figure 5K). Notably, the pDNA@E–H–E′ + H_2_O_2_ group exhibited the most pronounced suppression of fibroblast proliferation after a 5 days coculture period (Figure 5K). In summary, these results indicated that the ROS‐triggered release of pDNA@PEI─PBA polyplexes from pDNA@E–H–E′ effectively repressed the fibroblast proliferation and adhesion.

To further investigate the effects of pDNA@E–H–E′ composite antiadhesion membrane on myofibroblast activation and collagen synthesis in vitro, the expressions of fibrosis‐associated genes in 208F cells among all groups were evaluated using q‐reverse transcriptase‐polymerase chain reaction (qRT‐PCR) assay. As illustrated in Figure 5H–J, the expression levels of α‐SMA, Col I, and Col III in the pDNA@E–H–E′ + H_2_O_2_ group were significantly lower than those in the other groups, demonstrating that the ROS‐triggered release of pDNA@PEI─PBA polyplexes from pDNA@E–H–E′ combined with the overexpression of IL‐37 in cells contributed to the inhibition of myofibroblast activation and collagen synthesis in 208F cells.

### 2.7 Gross Observation and Histological Assessments

**Figure 6:**
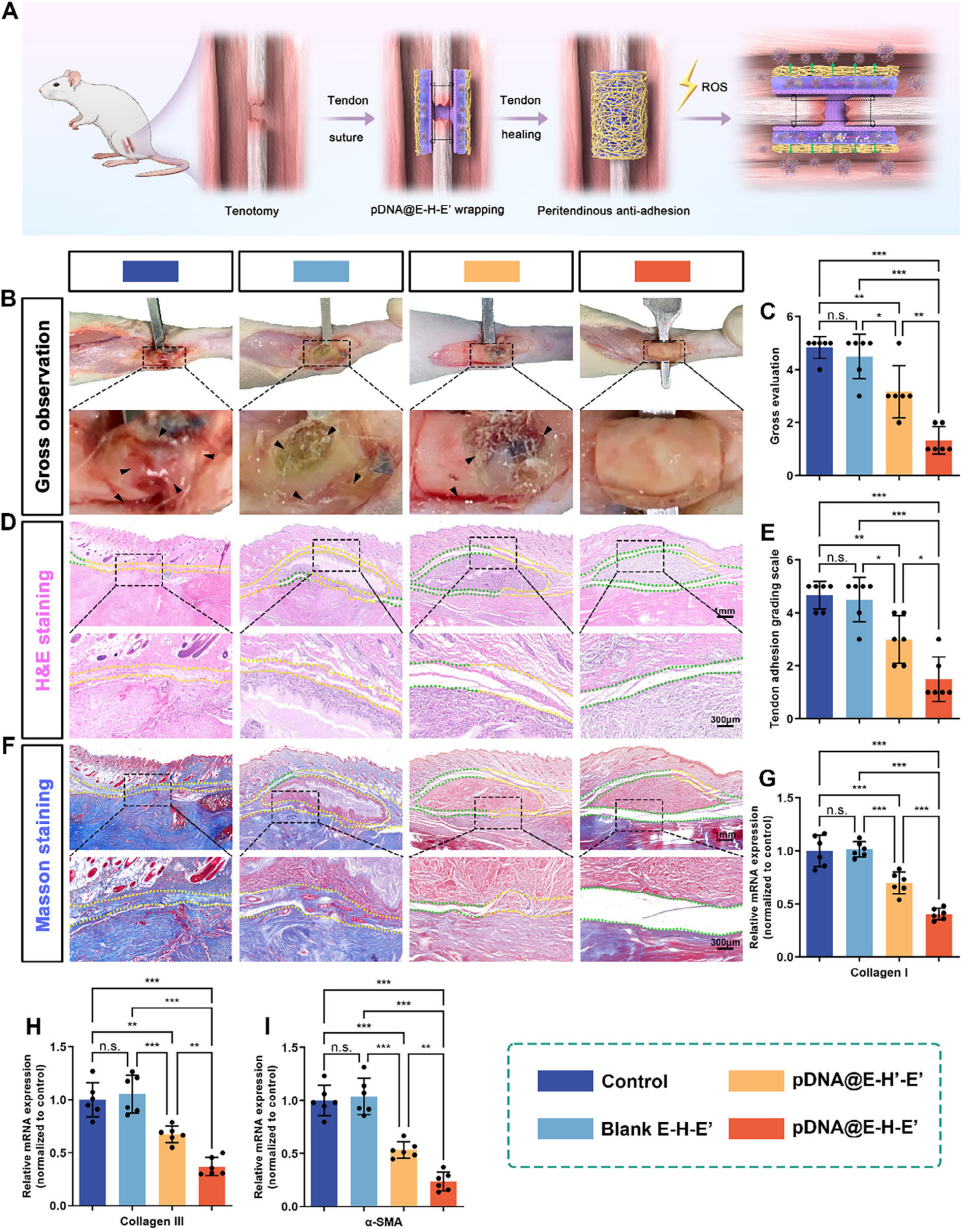
Peritendinous antiadhesion effects of pDNA@E–H–E′ after tendon injury in vivo. A) Schematic illustration of establishment of rat Achilles tendon adhesion model and application of pDNA@E–H–E′ for peritendinous antiadhesion. B) Gross observation of tendon adhesion at the repaired sites wrapped with no membranes, Blank E–H–E′, pDNA@E–Hʹ–Eʹ, and pDNA@E–H–E′ membranes at 21 days postoperatively (black arrows: the adhesion areas). C) The gross scores of peritendinous adhesion. Representative D) H&E and F) Masson staining images from different groups (green areas: the physiological peritendinous spaces; yellow areas: the adhesion tissues). E) The histologic scores of peritendinous adhesion. qRT‐PCR analysis of mRNA expressions of G) Col I, H) Col III, and I) α‐SMA in peritendinous adhesion tissues (mean ± SD, *p < 0.05, **p < 0.01, ***p < 0.001, *n* = 6).

To investigate the peritendinous antiadhesion effects of pDNA@E–H–E′ at the repair sites, we wrapped various antiadhesion materials onto the ruptured tendons. At 21 days postoperation, the Achilles tendon surgical sites were exposed and peritendinous adhesion was evaluated by direct observation (Figure **6**B). In the control group without treatment using antiadhesion membranes, the areas between the ruptured tendon and peritendinous tissues were filled with extensive adhesive tissues, which was difficult for blunt dissection. In the group receiving Blank E–H–E′ treatment, there were still dense bundles of adhesion tissues observed on the repair sites. In the groups receiving pDNA@E–Hʹ–Eʹ and pDNA@E–H–E′ treatments, the amount of adhesion tissues was significantly reduced, suggesting that the antiadhesion effects of IL‐37 was achieved by the release of pDNA@PEI─PBA nanocomposites. The surface of ruptured tendon was still occupied by tiny adhesion tissues in the group receiving pDNA@E–Hʹ–Eʹ treatment. Notably, almost no dense adhesion tissues were detected around the repaired sites wrapped with pDNA@E–H–E′, and the peritendinous areas could be easily released by blunt separation (Figure 6B). The adhesion scores from gross observations were summarized in Figure 6C, and the scores of the pDNA@E–H–E′ group were the lowest among the four groups.

Representative hematoxylin–eosin (H&E) and Masson's trichrome staining of histological sections of the repaired Achilles tendon and peritendinous tissues among four groups were shown in Figure 6D,F. The green areas referred to the physiological peritendinous spaces and the yellow areas referred to the adhesion tissues. Both in the control and Blank E–H–E′ groups, the peritendinous areas were filled with abundant adhesion tissues, and even part of adhesion tissues invaded into the disrupted tendon. In the groups receiving pDNA@E–Hʹ–Eʹ and pDNA@E–H–E′ treatments, the areas of physiological peritendinous intervals remarkably increased, and especially few adhesive tissues were observed on the surface of the repaired sites wrapped with pDNA@E–H–E′ membranes. Figure 6E indicated the adhesion scores based on histological findings and the pDNA@E–H–E′ treatment significantly lowered the adhesion grade of ruptured tendon.

### 2.8 Collagen III Deposition, ROS Levels, Gene and Protein Expressions in Peritendinous Tissues

**Figure 7:**
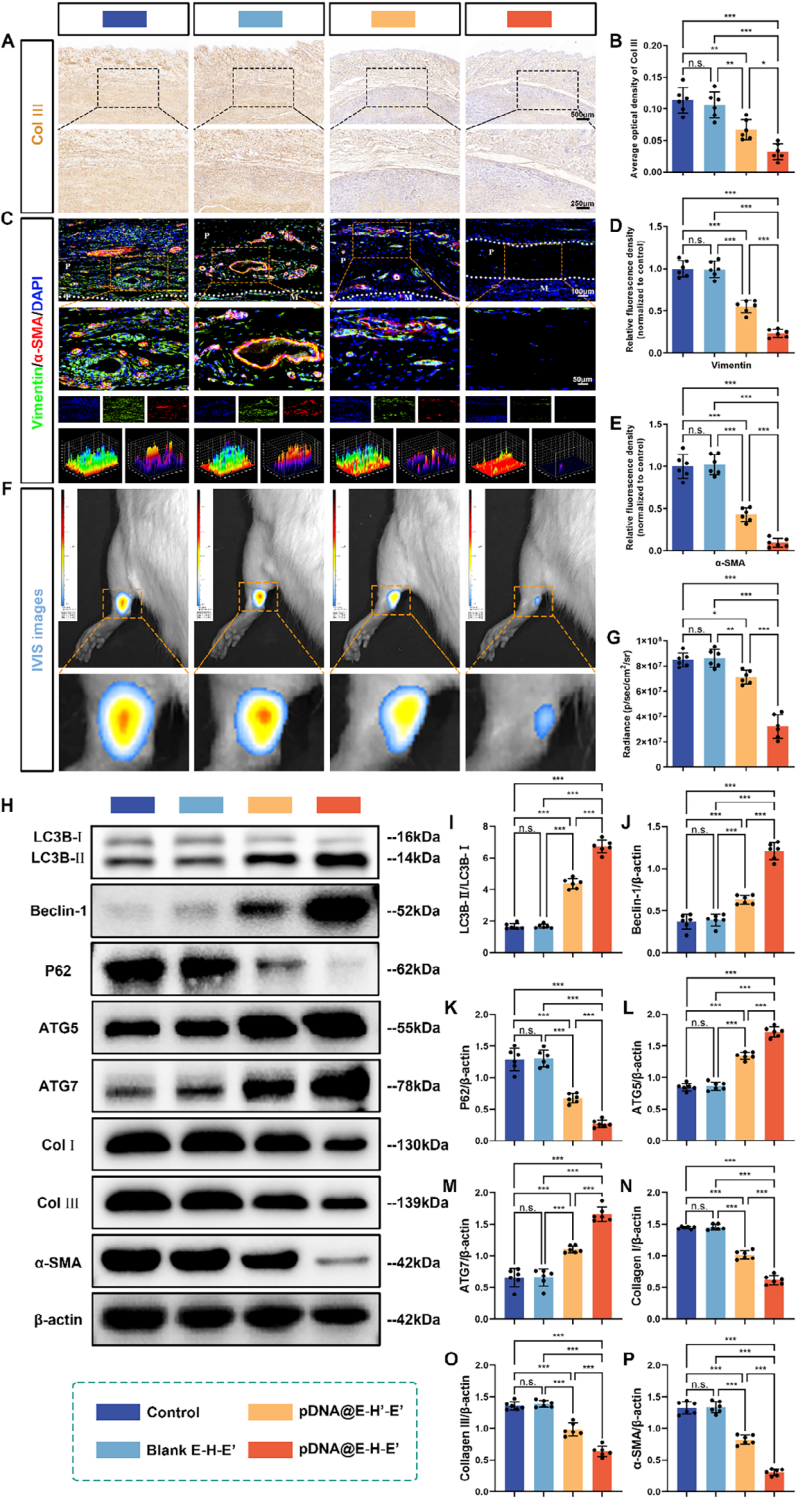
Fibrosis, autophagy, and ROS levels in adhesion tissues after treatment with pDNA@E–H–E′. A) Representative immunohistochemical staining images depicting Collagen III expression in peritendinous adhesion tissues, and B) the corresponding quantitative analysis of average optical density of Collagen III across different groups. C) Representative immunofluorescence images showing costaining of Vimentin (green) and α‐SMA (red) in the peritendinous adhesion tissues from different groups. Semiquantitative analysis of fluorescence intensity for D) Vimentin and E) α‐SMA. ROS levels in the peritendinous adhesion tissues after various treatments were indicated by F) representative IVIS images and G) the corresponding statistical analysis. H) Protein expressions of key markers associated with autophagy (LC3B, Beclin‐1, P62, ATG5, and ATG7) and fibrosis (Collagen I, Collagen III, and α‐SMA) in the peritendinous adhesion tissues after various treatments were determined by western blotting. I) LC3B, J) Beclin‐1, K) P62, L) ATG5, M) ATG7, N) Collagen I, O) Collagen III, and P) α‐SMA expressions were normalized to β‐actin (mean ± SD, *p < 0.05, **p < 0.01, ***p < 0.001, *n* = 6).

As the main component of collagen fibers during the formation of adhesion tissues, Col III expression in peritendinous adhesion tissues among the four groups was evaluated by immunohistochemistry staining (Figure **7**A). Dense Col III positive fibers stained brownish‐yellow were detected in peritendinous adhesion tissues in the control and Blank E–H–E′ groups, whereas Col III expression was observably decreased around the repaired sites wrapped with pDNA@E–H–E′ (Figure 7A). As shown in Figure 7B, the average optical density of Col III in pDNA@E–H–E′ group was the lowest among the four groups, indicating that overexpression of IL‐37 inhibited the expression of downstream Col III.

The effects of pDNA@E–H–E′ composite antiadhesion membrane on tendon adhesion formation were also investigated by detecting the expressions of fibrosis‐associated genes in peritendinous adhesion tissues using qRT‐PCR assay. Figure 6G–I demonstrated that the pDNA@E–H–E′ treatment group significantly decreased the expression levels of Col I, Col III, and α‐SMA compared to the other three groups. This observation suggested that pDNA@E–H–E′ achieved superior therapeutic efficacy against tendon adhesion through two synergistic mechanisms: the ROS‐triggered release of pDNA@PEI─PBA polyplexes from the composite membrane, coupled with the overexpression of IL‐37 in peritendinous tissues. Furthermore, immunofluorescence double‐staining analysis of Vimentin (green fluorescence) and α‐SMA (red fluorescence) in the peritendinous adhesion tissues provided additional experimental validation, thereby corroborating the aforementioned conclusions (Figure 7C‐E). As a type III intermediate filament protein, Vimentin serves as a specific marker of fibroblasts and plays a pivotal role in the regulation of tissue fibrogenesis in response to injury by regulating cell migration, extracellular matrix remodeling, and fibroblast‐to‐myofibroblast transition during tissue repair processes.^[^ ^56^ ^]^ Consequently, enhanced green fluorescence intensity serves as a reliable indicator of severe tissue fibrogenesis. α‐SMA, a well‐established marker of myofibroblasts, is a vital effector in tissue fibrosis and neovascularization by driving myofibroblast activation, extracellular matrix deposition, and the stabilization of nascent blood vessels during pathological tissue repair.^[^ ^57^, ^58^ ^]^ Therefore, the reinforcement in red fluorescence intensity signifies increased fibrosis and capillary angiogenesis within the tissues. As depicted in Figure 7C–E, the expression levels of Vimentin and α‐SMA in the pDNA@E–H–E′ treatment group were notably lower than those in the other three groups, highlighting the outstanding antifibrotic efficacy of the pDNA@E–H–E′ composite antiadhesion membrane.

To further investigate the underlying regulatory mechanisms by which pDNA@E–H–E′ composite antiadhesion membrane suppressed adhesion tissues formation, western blotting assay was conducted to detect the expressions of autophagy‐associated proteins, including LC3B, Beclin‐1, P62, ATG5, and ATG7, fibrosis‐associated proteins, including α‐SMA, Col I, and Col III, in peritendinous adhesion tissues. As illustrated in Figure 7H–P, the groups administered with pDNA@E–Hʹ–Eʹ and pDNA@E–H–E′ exhibited a substantial upregulation of autophagy‐related markers, including LC3B, Beclin‐1, ATG5, and ATG7, when compared to both the control group and Blank E–H–E′ group. By contrast, the expression levels of P62 and fibrotic markers, specifically α‐SMA, Col I, and Col III, were markedly reduced in the pDNA@E–Hʹ–Eʹ and pDNA@E–H–E′ treatment groups relative to the other two groups. These findings collectively demonstrated that the upregulation of IL‐37 expression in peritendinous tissues played a crucial role in promoting autophagy activities while simultaneously inhibiting the formation of adhesion tissues, primarily through an autophagy‐mediated regulatory mechanism. More importantly, in comparison with the pDNA@E–Hʹ–Eʹ group, the pDNA@E–H–E′ treatment demonstrated superior therapeutic effects by significantly upregulating the expressions of autophagy‐related markers (LC3B, Beclin‐1, ATG5, and ATG7) while simultaneously downregulating the levels of P62 and fibrosis‐associated markers (α‐SMA, Col I, and Col III) in peritendinous tissues. These findings suggested that the on‐demand release mechanism of pDNA@PEI─PBA polyplexes from the pDNA@E–H–E′ composite antiadhesion membrane contributed to induction and maintenance of autophagy activities, thereby resulting in enhanced therapeutic efficacy against tendon adhesion formation (Figure 7H–P).

One potential mechanism underlying the pathogenesis of tendon adhesion may involve oxidative stress in peritendinous tissues, driven by the excessive production of ROS.^[^ ^32^ ^]^ To assess the in vivo ROS scavenging capability of the pDNA@E–H–E′ composite antiadhesion membrane, L‐012, a ROS‐specific bioluminescent probe, was employed to measure ROS levels using IVIS imaging (Figure 7F,G). Prominent bioluminescent signals were observed in both the control group and the Blank E–H–E′ group, suggesting significantly elevated ROS levels under tendon adhesion conditions. While the pDNA@E–Hʹ–E′ treatment partially decreased local ROS levels, the reduction was insufficient to achieve an optimal effect. Importantly, the pDNA@E–H–E′ treatment led to a significant reduction in ROS levels, potentially attributed to the following mechanisms: first, borate ester bonds between PVA─MA hydrogel and pDNA@PEI─PBA polyplexes exhibit specific reactivity toward ROS, through a dynamic cleavage mechanism, thereby serving as effective ROS scavengers in oxidative stress modulation;^[^ ^59^, ^60^ ^]^ second, the upregulation of IL‐37 expression significantly reduces ROS levels, potentially through its anti‐inflammatory properties and its ability to modulate key signaling pathways such as NF‐κB and MAPK, which are closely associated with oxidative stress.^[^ ^61^, ^62^ ^]^

### 2.9 Histological Evaluation of Tendon Healing Status

**Figure 8:**
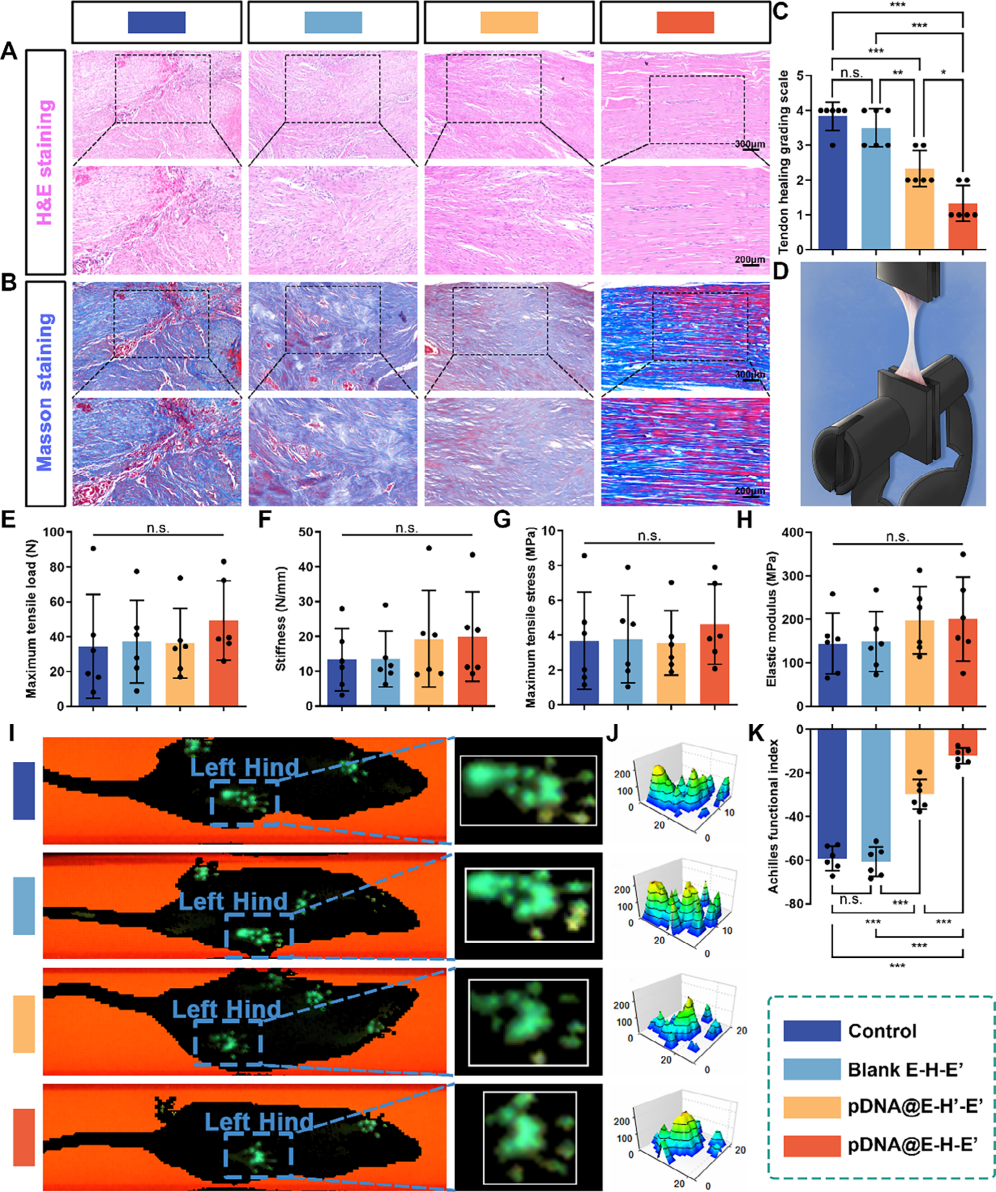
pDNA@E–H–E′ promoted the scarless repair of Achilles tendon and ameliorated Achilles tendon motor function. Representative A) H&E and B) Masson staining images of tendon tissues at the repaired sites wrapped with no membranes, Blank E–H–E′, pDNA@E–Hʹ–Eʹ, and pDNA@E–H–E′ membranes at 21 days postoperatively. C) The histologic scores of tendon healing. D) Schematic diagram of mechanical testing of the rat Achilles tendon. Statistical analysis of E) maximum tensile load, F) stiffness, G) maximum tensile stress, and H) elastic modulus of the repaired tendons from different groups. I) Gait analysis and footprints of experimental rat from different groups. J) The 3D footprint intensities of rat left hind limbs from different groups. K) Achilles function index (AFI) assessment of the repaired tendons from different groups at 21 days postoperatively (mean ± SD, *p < 0.05, **p < 0.01, ***p < 0.001, *n* = 6).

H&E and Masson's trichrome staining of the repaired tendons were performed to assess the qualities of tendon healing. As illustrated in Figure **8**A,B, irregularly arranged collagen fiber bundles with partial disruption, accompanied by excessive adhesion tissue ingrowth, were observed in the repaired tendons of both the control group and the Blank E–H–E′ group. By contrast, compared to the other three groups, the pDNA@E–H–E′ treatment group exhibited superior tendon healing outcomes, as evidenced by well‐organized, densely packed collagen fiber bundles and improved tendon continuity (Figure 8A,B). Furthermore, histological scoring based on tendon healing quality revealed that the pDNA@E–H–E′ treatment group achieved the lowest score among the four groups, demonstrating superior tendon healing outcomes compared to the other three groups (Figure 8C).

### 2.10 Assessment of Tendon Biomechanical Properties and Motor Function

Gait recovery and locomotion performance serve as essential postoperative functional assessment metrics for evaluating tendon repair outcomes.^[^ ^63^ ^]^ The Achilles functional index (AFI) was measured and analyzed to assess the locomotion performance of repaired tendons, with more negative AFI values correlating with increased severity of mobility impairment. As illustrated in Figure 8I–K, the hind footprints on the repaired side in both the control group and the Blank E–H–E′ group were noticeably longer and narrower compared to the normal side, and their AFI values were the most negative among all groups, reflecting the most severe postoperative mobility impairment. Notably, the pDNA@E–H–E′ treatment group demonstrated significantly elevated AFI values when compared to the other three groups (Figure 8I–K). This observation suggested that the pDNA@E–H–E′ composite antiadhesion membrane effectively improved Achilles tendon motor function through the inhibition of tendon adhesion and tendon scar repair, highlighting its promising potential for the treatment of tendon injuries.

As shown in Figure 8E–H, to further assess tendon healing strength and investigate whether the pDNA@PEI─PBA polyplexes released from the pDNA@E–H–E′ membrane interfere with the natural tendon healing process, we conducted a systematic evaluation of key biomechanical parameters, including maximum tensile load, stiffness, maximum tensile stress, and elastic modulus, of the regenerated tendons. The results revealed that the strength–tensile displacement curves of the four groups displayed a similarly upward trend, with no significant differences observed in the biomechanical properties of the regenerated tendons between the groups (Figure 8E–H and Figure S14 (Supporting Information)). These findings indicated that minimizing tendon adhesions through suppression of the exogenous healing process did not compromise the intrinsic mechanical strength of the tendon. Enhancing the preservation of the tendon's endogenous healing process offered a promising strategy for achieving adhesion‐free tendon regeneration.

### 2.11 Translational Potential of pDNA@E–H–E′ Composite Barrier

More importantly, the long‐term stability of the barrier in vivo and the potential off‐target effects of IL‐37 overexpression are critical considerations for translational relevance of the pDNA@E–H–E′ barrier.

The pDNA@E–H–E′ barrier was designed to balance durability with controlled degradation to support tendon repair while preventing adhesion formation. The pDNA@E–H–E′ barrier, composed of biocompatible polymers, provide mechanical stability. In particular, the outer (Eʹ) and inner (E) electrospun layers are composed of biodegradable PLA and PCL, respectively, which are known for the slow degradation profile and mechanical robustness. These properties ensure that the barrier maintains its structural integrity during the critical early and intermediate phases of tendon healing (up to several weeks postinjury), providing sustained mechanical support during tendon repair. Our in vivo histological and macroscopic assessments at 3 weeks postimplantation confirmed that the barrier maintained structural integrity and remained adherent to the tendon surface without evidence of premature degradation or dislocation, effectively preventing fibroblast infiltration and adhesion formation without eliciting significant inflammatory responses (Figure 6B,D,F). These findings indicate that the barrier's stability is well‐suited for the therapeutic window of tendon repair.

To minimize the risk of off‐target gene expression, we implemented a unidirectional delivery strategy using the Eʹ–H–E configuration. The PCL‐EFM with lower porosity as the inner layer (E) facing the tendon surface functions as a diffusion barrier to restrict the migration of pDNA toward the tendon, thereby preventing unwanted modulation of tendon‐resident cells. IL‐37 expression was confirmed to be localized predominantly in the peritendinous region, as evidenced by Figure 5B,D and Figure S8 (Supporting Information). Moreover, IL‐37 is an endogenous anti‐inflammatory cytokine with a well‐established safety profile. Its overexpression has not been associated with profibrotic or cytotoxic effects in previous reports. In our study, we observed no histopathological signs of aberrant tissue remodeling, fibrosis, or immune dysregulation in surrounding organs such as the heart, liver, spleen, lung, and kidney (Figure S15, Supporting Information).

In summary, our design minimizes systemic exposure while enabling site‐specific therapeutic effects. Nonetheless, further preclinical evaluation in large animal models and extended follow‐up studies are essential steps toward clinical translation.

## 3 Conclusion

This study demonstrates that dysregulated autophagy plays a significant role in tendon adhesion formation following tendon injury. Our findings suggest that autophagy may exert a protective effect, inhibiting the formation of tendon adhesions, and that IL‐37 could be a potential therapeutic target for preventing tendon adhesions through autophagy activation. The development of the novel three‐layer composite antiadhesion barrier, pDNA@E–H–E′, which facilitates on‐demand and unidirectional delivery of bioactive pDNA for IL‐37 overexpression, offers an innovative strategy for tendon adhesion prevention. Through the localized delivery of IL‐37‐encoding pDNA nanocomposites, the research underscores the crucial role of autophagy in the prevention of excessive fibroblast proliferation, FMD, and collagen synthesis. In vivo, the application of pDNA@E–H–E′ membranes in a rat Achilles tendon adhesion model significantly inhibited peritendinous adhesion formation and promoted scarless repair of Achilles tendon, further confirming the barrier's therapeutic efficacy. Overall, this study highlights the potential of autophagy activation through IL‐37 overexpression as a promising approach to prevent tendon adhesions, and introduces the three‐layer composite antiadhesion barrier (pDNA@E–H–E′) for tendon adhesion prevention, offering a novel and efficient strategy for tendon repair.

## 4 Experimental Section

### Fabrication, Characterization, and Transfection Efficacy of pDNA@PEI─PBA Polyplexes

The synthesis and characterization of PEI─PBA were conducted according to the previous studies.^[^ ^64^ ^]^ PEI─PBA and pDNA were dissolved in diethylpyrocarbonate water and then combined at varying mass ratios [*M* = *M*(PEI─PBA)/*M*(pDNA)] to form gene transfection polyplexes. The particle size distribution and zeta potentials of pDNA@PEI─PBA polyplexes at different mass ratios were measured by DLS using Malvern Zetasizer Nano ZS90 (Malvern, UK). The morphology of pDNA@PEI─PBA polyplexes was further examined through TEM with a JEM 2100F system. To conduct in vitro transfection of pDNA@PEI─PBA polyplexes, rat fibroblast 208F cell lines were treated as a cell model and cultured in Dulbecco's modified Eagle Medium (DMEM) supplemented with 10% FBS and 1% antibiotics at 37 °C in a 5% CO_2_ humidified incubator. The cells were seeded into 24‐well plates and allowed to grow for 24 h. Subsequently, the medium was changed to 100 µL of pDNA@PEI─PBA polyplexes solution and 400 µL of DMEM medium in each well. After a 6 h incubation, the transfection medium was removed and replaced with 500 µL of DMEM supplemented with 10% FBS. The cells were then maintained for an additional 72 h and examined under a fluorescence microscope (DMi8, Leica Microsystems Co., Ltd.). Transfection efficiency was assessed via quantitatively analyzing the proportion of eGFP‐positive cells using a flow cytometer (BD FACSCalibur). On the fifth day after 208F cell transfection, the culture supernatant was harvested and centrifuged at 4 °C for 15 min. The secretion of IL‐37 in supernatant after 208F cells transfection was determined by the IL‐37 ELISA Kit (AdipoGen).

### Effects of pDNA@PEI─PBA Polyplexes on TGF‐β1‐Induced Fibroblast Proliferation, FMD, and Collagen Synthesis

Cell proliferation of 208F cells after various treatments was investigated using CCK‐8 (Dojindo Molecular Technologies, Inc., Japan) assay in accordance with the manufacturer's protocol. The results were expressed using cell proliferation rate by normalizing the average absorbance values to the control group. Additionally, FCM was performed to analyze cell cycle distribution, providing further insight into the proliferation of 208F cells under various treatments. Western blotting assay was performed to detect the expressions of autophagy‐associated proteins, including LC3B, Beclin‐1, P62, ATG5, and ATG7,^[^ ^65^ ^]^ fibrosis‐associated proteins, including Collagen I, Collagen III, and α‐SMA^[^ ^66^ ^]^ in 208F cells among all groups, with β‐actin serving as the internal control. LC3B and α‐SMA expressions in 208F cells following various treatments were also assessed by immunofluorescence staining. After fixation, permeabilization, and blocking, cultured cells were incubated overnight at 4 °C with the primary antibodies against α‐SMA (Abcam, ab7817, 1:100) and LC3B (Abcam, ab63817, 1:100). After washing thrice with phosphate‐buffered saline (PBS), the samples were incubated with appropriate fluorescent secondary antibodies for 1 h and then stained with DAPI (1 mg mL^−1^) for 10 min at room temperature. Finally, the cells were observed and imaged using a fluorescence microscope (DMi8, Leica Microsystems Co., Ltd.).

### Preparation and Characterization of pDNA@E–H–E′ Composite Membranes

First, the PCL‐EFM (E) was fabricated according to the electrospinning parameters and procedures as previously described.^[^ ^67^ ^]^ Briefly, for preparing PCL electrospinning solution, 2 g PCL (average *M*_n_ ≈80 kDa) was completely dissolved in a mixed solvent of 6 g dichloromethane and 4 g *N*,*N*‐dimethylformamide. The electrospinning solution was loaded into a 10 mL syringe, and drawn from the needle tip to the collector by the electrostatic force to produce PCL‐EFM. Then, PVA─MA was synthesized through reactions between the hydroxyl groups on the PVA and the epoxy groups on the methacrylate glycidyl ether under the dimethyl sulfoxide as solvent, as described before.^[^ ^68^ ^]^ Subsequently, according to the previous studies, the preparation of PLA/gelatin short‐fiber EFM (E′) involved several key steps: 1) preparing PLA/gelatin electrospinning solution (12 wt%) by dissolving PLA and gelatin in hexafluoroisopropanol at a mass ratio of 1:4; 2) obtaining PLA/gelatin nanofiber meshes under appropriate electrospinning conditions; 3) cutting the nanofibers into small pieces; 4) fully dispersing the small pieces in *tert*‐butanol by homogenizing with IKA T‐18 at 13 000 rpm for 15 min; 5) freeze‐drying the samples to obtain the un‐cross‐linked membranes; 6) cross‐linking the membranes using thermal cross‐linking at 180 °C for 2 h in an oven to stabilize the structures.^[^ ^69^ ^]^ Finally, the pDNA@PEI─PBA polyplexes were mixed with PVA─MA hydrogel solutions, and the obtained mixture (H) was uniformly dispersed into the gaps between horizontal placed PCL‐EFM and PLA/gelatin short‐fiber EFM followed by irradiation at 405 nm blue light (8 mW cm^−2^) to form pDNA@E–H–E′ composite antiadhesion membranes.

^1^H NMR spectroscopy was performed using a Bruker 600 MHz spectrometer (Germany) with deuterated dimethyl sulfoxide as the solvent. The morphologies of PLA/gelatin short‐fiber EFM, PCL‐EFM, pDNA@PEI─PBA polyplexes‐loaded PVA─MA hydrogel, and pDNA@E–H–E′ composite membranes were observed using SEM. The surface wettability of various membranes was examined using a water contact angle analyzer (DSA25S; Data Physics Corporation) at room temperature. The stress–strain curves of PLA/gelatin short‐fiber EFM, PCL‐EFM, and pDNA@E–H–E′ composite membranes were measured using an MTS Exceed E42 universal tensile testing machine. The maximum test stress was set to 100 N, with a tensile speed of 50 mm min^−1^. Samples were prepared with a uniform width of 2 mm, thickness of 0.05 mm, and a gauge length (standard distance) of 20 mm. Young's moduli were calculated based on the tensile stress–strain curves obtained from mechanical testing.

### In Vitro Polyplexes ROS‐Responsive and Unidirectional Release Study

The unidirectional release property of pDNA@E–H–E′ composite membranes was determined by using Transwell plates to analyze pDNA@PEI─PBA polyplexes release from both two sides of pDNA@E–H–E′ (Figure 5A). Briefly, the PLA/gelatin short‐fiber‐EFM (E′) side and PCL‐EFM (E) side of pDNA@E–H–E′ composite membranes, which were immersed in PBS at pH 7.4 with 0.1 mm H_2_O_2_ at 37 °C, were placed at the bottom of the upper chamber. At the predetermined time points, 1 mL of solution was collected from the lower chamber and stored at −20 °C for subsequent analysis, while an equal volume of fresh PBS containing H_2_O_2_ was added. The fluorescein‐isothiocyanate‐labeled pDNA was used to monitor the release process (*λ*_ex_ = 494 nm, *λ*_em_ = 520 nm) and the released nanocomposite contents were quantified by detecting fluorescence of the harvested samples. Moreover, 208F cells were seeded in the lower chamber of 24‐well Transwell plates, while the Eʹ‐side or E‐side of pDNA@E–H–E′ composite membranes was positioned at the base of the upper chamber to facilitate coculture with the cells. After coculturing for 1, 3, and 5 days, the cells were examined using a fluorescence microscope, and the transfection efficiency was quantified by analyzing the percentage of eGFP‐positive cells using a flow cytometer (BD FACSCalibur).

In addition, to investigate the ROS‐triggered nanocomposites release behavior of composite antiadhesion membranes, the pDNA@E–Hʹ–Eʹ and pDNA@E–H–E′ membranes were incubated in PBS (pH = 7.4) with or without 0.1 mm H_2_O_2_ and maintained in a shaker at 37 °C (Hʹ: non‐ROS‐responsive PVA─MA hydrogel loaded with pDNA@PEI polyplexes; H: ROS‐responsive PVA─MA hydrogel loaded with pDNA@PEI─PBA polyplexes). 1 mL supernatant was collected at the predetermined time points and stored at −20 °C before analysis while replenishing with equal PBS with or without H_2_O_2_. The released nanocomposite contents were quantified by detecting fluorescence of the harvested samples.

### Evaluation of Anticell Adhesion, Fibrosis‐Associated Gene Expressions, Cell Proliferation Inhibition of pDNA@E–H–E′ In Vitro

Rat fibroblast 208F cell lines, applied as a cell model, were seeded on various membranes in 24‐well culture plates for morphological observation, proliferation assay, and evaluation of gene expression. Antiadhesion capability of antiadhesion membranes was evaluated by observation of cytoskeletal arrangements after 3 days of coculture. In brief, 208F cells on the respective membranes were first fixed in 4% paraformaldehyde for 20 min, flushed repeated by PBS, and permeabilized with 0.1% Triton X‐100 for 10 min. Subsequently, the cytoskeleton and cell nucleus were stained with rhodamine‐labeled phalloidin and DAPI, respectively, following the manufacturer's protocol. The specimens were then examined using a fluorescence microscope, and average spreading areas of 208F cells on each surface were analyzed by the ImageJ software. The proliferation of 208F cells on various membranes was assessed on days 3 and 5 using the CCK‐8 assay, following the manufacturer's protocol. The qRT‐PCR assay was conducted to evaluate the expressions of fibrosis‐related genes, including Collagen I, Collagen III, and α‐SMA, in 208F cells among all groups. Total RNA was extracted after 5 days of coculture using TRIzol Reagent (Invitrogen, USA), following the manufacturer's protocol. Then, complementary DNA was synthesized by means of 1 mg of RNA and Prime‐Script RT Master Mix (Takara). Gene expressions were detected with SYBR Green detection reagent (Takara) and ABI 7500 Sequencing Detection System (Applied Biosystems Inc, CA, USA). Relative mRNA levels were calculated using the 2^−ΔΔCT^ method and normalized to *GAPDH* as the internal reference gene. Primer sequences are provided in Table S1 of the Supporting Information.

### Rat Achilles Tendon Injury Model

Rat Achilles tendon injury model was chosen as the animal model of tendon adhesion in this study. All animal experiments were conducted in accordance with the guidelines of Institutional Animal Care and Use Committee of Shanghai Jiao Tong University (A2023222). In brief, after intraperitoneal anesthesia with sodium pentobarbital, the posterior limbs of Sprague‐Dawley rats (weighing 200–250 g) were sterilized. A tendon adhesion model was established by transversely transecting the Achilles tendon through posterior middle skin incision and repairing the ruptured tendon with 6‐0 polypropylene sutures by means of modified Kessler technique. A total of 48 rats were randomly divided into four groups (*n* = 12 per group). In the three experimental groups—Blank E–H–E′, pDNA@E–Hʹ–Eʹ, and pDNA@E–H–E′—1  ×  1.5 cm pieces of the respective composite membranes were wrapped around the sutured Achilles tendon, while the sutured tendon without treatments was taken as control.

### Macroscopic Evaluation and Histological Evaluation

Three weeks postoperation, the skins behind Achilles tendon were longitudinally incised to expose repaired tendon and peritendinous tissue. Tendon adhesion severity was assessed macroscopically using a standardized grading system, as described in the previous studies^[^ ^70^ ^]^ (Figure S10, Supporting Information).

The rats from each group were sacrificed and the hind limbs were transected and harvested. For the histomorphological observation, the samples were fixed in 4% paraformaldehyde for 48 h, decalcified in 10% ethylenediaminetetraacetic acid disodium salt for 2 months, and subsequently embedded in paraffin. Sagittal sections (5 µm thick) were prepared and stained with H&E, Masson's trichrome, and immunohistochemically labeled for type III collagen for histological evaluation. To histologically assess severity of tendon adhesion, a previously established histological scoring system was used for semiquantitative analysis, categorizing adhesion into five grades^[^ ^70^ ^]^ (Figure S11, Supporting Information). Additionally, tendon healing at the surgical site was assessed using a dedicated histological scoring system (Figure S12, Supporting Information).

### Double Labeling Immunofluorescence Staining

Paraffin‐embedded sections were dewaxed in fresh xylene, dehydrated through graded ethanol concentrations, and washed in PBS solution. 3% H_2_O_2_ solution and 10% normal goat serum were incubated onto the tissues for 30 min to eliminate endogenous peroxidase activity and block nonspecific binding sites, respectively. Subsequently, the primary antibodies against Vimentin (Abcam, ab92547, 1:2000) and α‐SMA (Abcam, ab7817, 1:2000) were applied dropwise to the target areas and incubated overnight at 4 °C. After washing, the samples were incubated with appropriate fluorescent secondary antibodies for 1 h, followed by visualization and imaging using a fluorescence microscope.

### qRT‐PCR and Western Blotting Analysis

Total RNA from peritendinous tissues was extracted, and qRT‐PCR assay was conducted, according to the methods mentioned above, to evaluate the expressions of fibrosis‐related genes, including Collagen I, Collagen III, and α‐SMA, in peritendinous tissues at the repaired sites from different groups.

Western blotting assay was carried out to assess the protein expressions of key markers associated with autophagy (LC3B, Beclin‐1, P62, ATG5, and ATG7) and fibrosis (Collagen I, Collagen III, and α‐SMA) in peritendinous adhesion tissues at the repaired sites from different groups. β‐actin served as the internal reference for protein loading normalization. Briefly, peritendinous adhesion tissues at the repaired sites from different groups were harvested. To isolate proteins from tissues, the tissues were homogenized in RIPA lysis buffer consisting of protease and phosphatase inhibitors. After homogenization, the lysates were centrifuged, and the resulting supernatants were collected. Protein concentrations in the supernatants were quantified using BCA assay. Equal amounts of protein samples (20 µg per lane) were separated by sodium dodecyl sulfate–polyacrylamide gel electrophoresis, electrotransferred onto polyvinylidene difluoride membranes, and blocked in 5% nonfat milk to reduce nonspecific binding. Then, the membranes were incubated with primary antibodies against LC3B (Abcam, ab192890, 1:2000), Beclin‐1 (Proteintech, 11306‐1‐AP, 1:2000), P62 (Abcam, ab109012, 1:10000), ATG5 (Proteintech, 10181‐2‐AP, 1:2000), ATG7 (Proteintech, 10088‐2‐AP, 1:1000), α‐SMA (Proteintech, 23081‐1‐AP, 1:2000), Col I (Proteintech, 14695‐1‐AP, 1:2000), Col III (Proteintech, 22734‐1‐AP, 1:1000), and β‐actin (Proteintech, 66009‐1‐Ig, 1:20000) overnight at 4 °C. Following a 2 h incubation at room temperature with horseradish‐peroxidase‐conjugated secondary antibodies (Proteintech), protein signals were detected using an enhanced chemiluminescence reagent (Epizyme, Shanghai, China) and visualized with the ChemiDoc CRS imaging system (Bio‐Rad, USA). For semiquantitative analysis, the relative protein expression was calculated in the form of relative gray level using ImageJ software.

### Detection of IL‐37 and ROS Concentrations in Peritendinous Tissues

Peritendinous tissues at the surgical sites from different groups were harvested. Following grinding, sonication, homogenization, and centrifugation, the resulting tissue homogenates were obtained and stored at −80 °C until analysis. IL‐37 concentrations in peritendinous tissues were determined by IL‐37 ELISA Kit (Adipogen), following the manufacturer's protocol. ROS concentrations in peritendinous tissues were determined using ROS Assay Kit (Cell Biolabs), following the manufacturer's protocol.

### Evaluation of ROS Scavenging Ability In Vivo

Prior to euthanasia, each rat received a single tail vein injection of the L‐012 probe (75 mg kg^−1^, Wako Chemical). Luminescence images were then captured using the IVIS imaging system (Lumina K Series III, PerkinElmer). All in vivo imaging procedures were performed under general anesthesia induced by pentobarbital sodium.

### Biomechanical Testing

To evaluate the tendon healing quality, the maximum tensile load, stiffness, maximum tensile stress, and elastic modulus of repaired tendon were evaluated across the four groups using a rheometer (Instron 5569; Instron, Norwood, MA, USA). Briefly, the proximal and distal ends of the repaired Achilles tendon were secured with force gauge clamps. The proximal end was then subjected to uniaxial traction at a constant rate of 20 mm min^−1^ until tendon failure, and the resulting strength–tensile displacement curves were recorded for analysis.

### Gait Analysis

At 21 days after surgery, functional recovery of the repaired Achilles tendons was evaluated through gait analysis and calculation of the AFI, following protocols established in previous studies.^[^ ^63^ ^]^ Briefly, one week before the treatments, rats were first trained to traverse the runway in search of a reward at the opposite end of the runway. The rats were allowed to traverse the enclosed walkway, equipped with a high‐speed video camera which was positioned underneath the runway. This camera, assembled with 35 mm wide‐angle lens, was used to record the rat's footprints as it moved across the runway. For gait analysis, footprint parameters including print length (defined as the distance between the third toe and heel, PL), toe spreading length (defined as the distance between the first and fifth toes, TS), and intermediary toe spreading length (defined as the distance between the second and fourth toes, IT) were obtained with CatWalk XT 10.6 software (Noldus, The Netherlands) to calculate AFI values. Then, according to the difference between the experimental (injured) side (E) and the contralateral normal side (N), three footprint dimension factors (PLF, TSF, ITF) could be obtained according to the following equations: PLF = (NPL − EPL)/EPL, TSF = (ETS − NTS)/NTS, ITF = (EIT − NIT)/NIT. Finally, the AFI was calculated according to an established equation: AFI = 74 (PLF) + 161 (TSF) + 48 (ITF).

### Statistical Analysis

Results were represented as the mean ± standard deviation (SD). Comparison of differences between two groups was achieved by Student's *t*‐test and multiple comparisons were made using a one‐way ANOVA followed by Tukey's posthoc test. GraphPad Prism 9.0 and SPSS 22.0 software was used to perform the statistical analysis and *p*‐value < 0.05 was considered to represent a significant difference.

## Conflict of Interest

The authors declare no conflict of interest.

## Supporting information



Supporting Information

## Data Availability

The data that support the findings of this study are available from the corresponding author upon reasonable request.
