# Supplementary material for: The Composite Antiadhesion Barrier Facilitated Fibroblast Autophagy Activation for Tendon Repair
Source: Adv Sci (Weinh). 2025 Sep 11;12(41):e06204. doi: 10.1002/advs.202506204 (PMC12591193; doi:10.1002/advs.202506204)
Supplement: Supplementary file 1 — Supporting Information [file ADVS-12-e06204-s001.docx]

**Supporting Information**

**
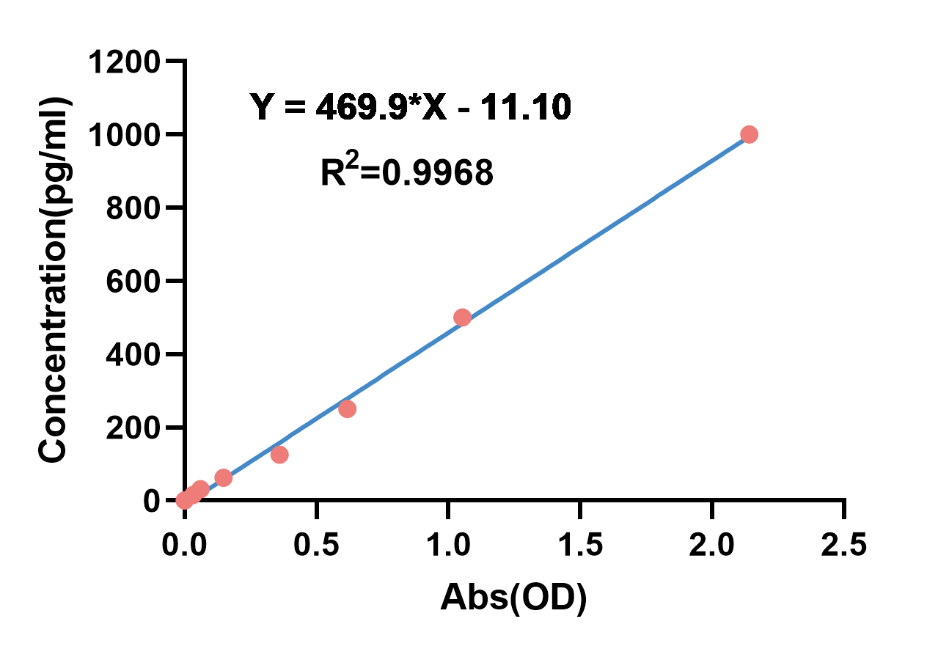
**

**Figure S1** Standard curve for absorbance and IL-37 concentration. The curve was drawn using linear regression in GraphPad Prism 9.

**
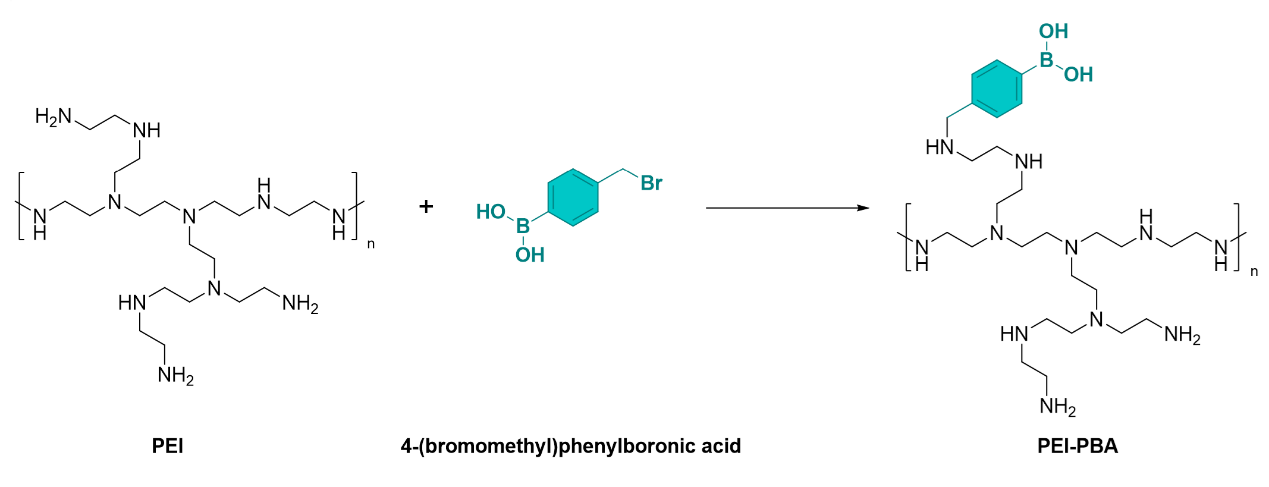
**

**Figure S2** Schematic diagram of the PEI-PBA synthesis route.

**
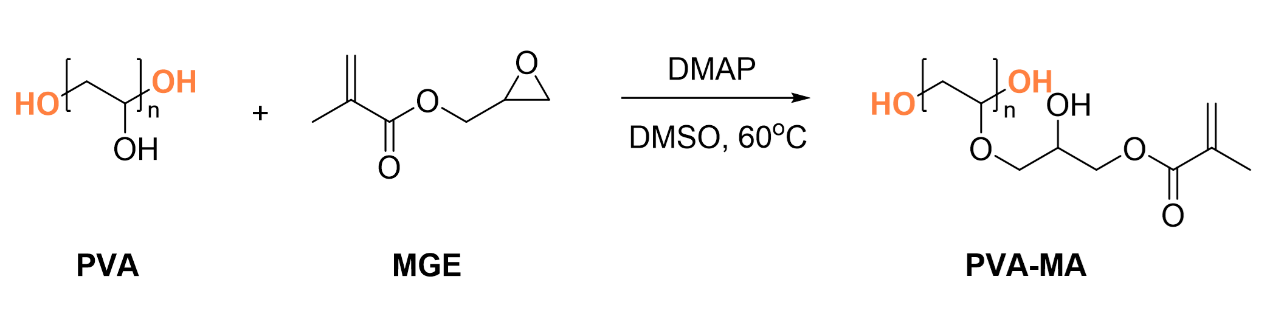
**

**Figure S3** Schematic diagram of the PVA-MA synthesis route.

**
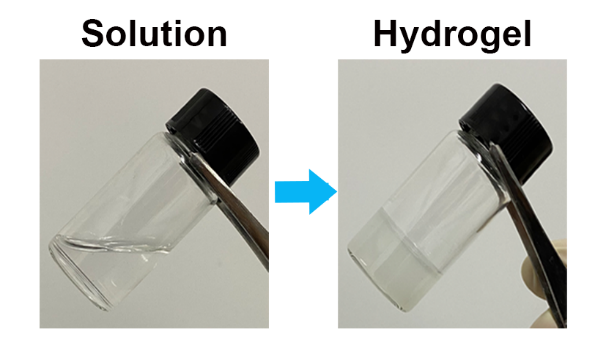
**

**Figure S4** Photographs of the solution to hydrogel transition under 405 nm ultraviolet irradiation.


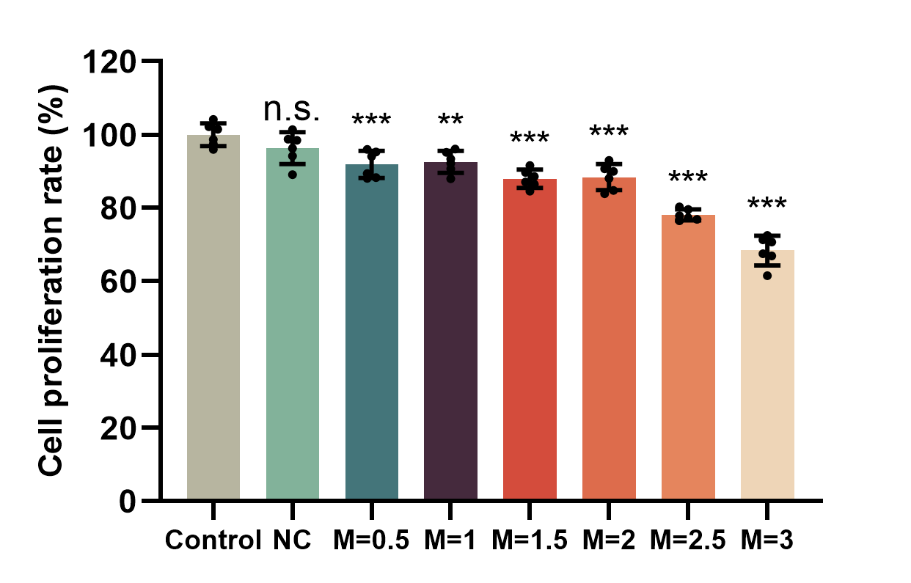


**Figure S5** Cytotoxicity of pDNA@PEI-PBA polyplexes at different *M* ratios. (mean ± SD, *p < 0.05, **p < 0.01, ***p < 0.001, n = 6)


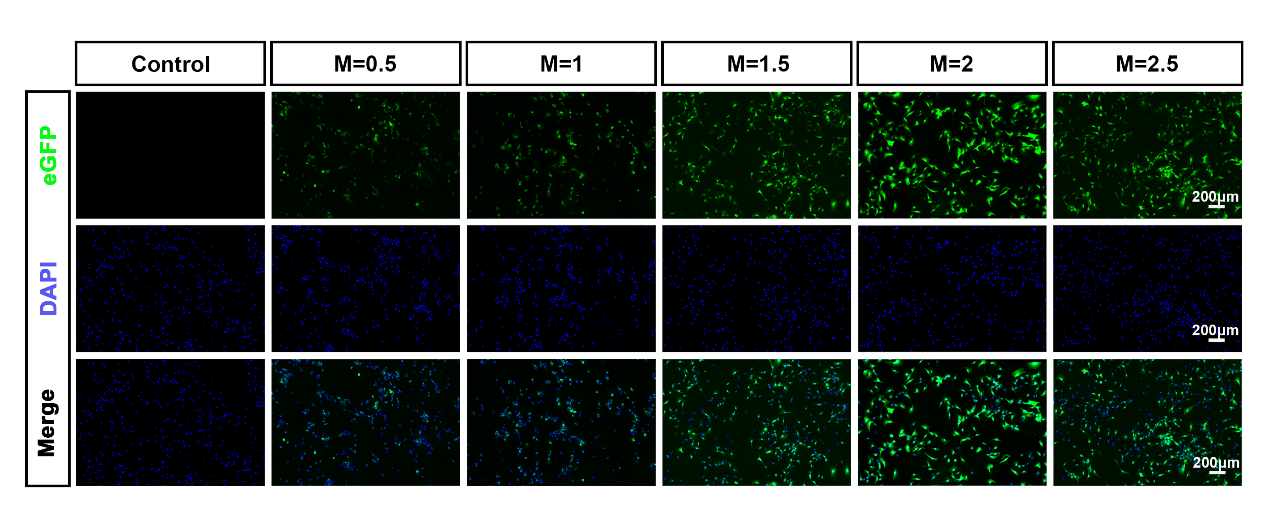


**Figure S6** Transfection efficiency of pDNA@PEI-PBA polyplexes at different *M* ratios for rat fibroblast 208F cell lines was determined by fluorescence microscopy.


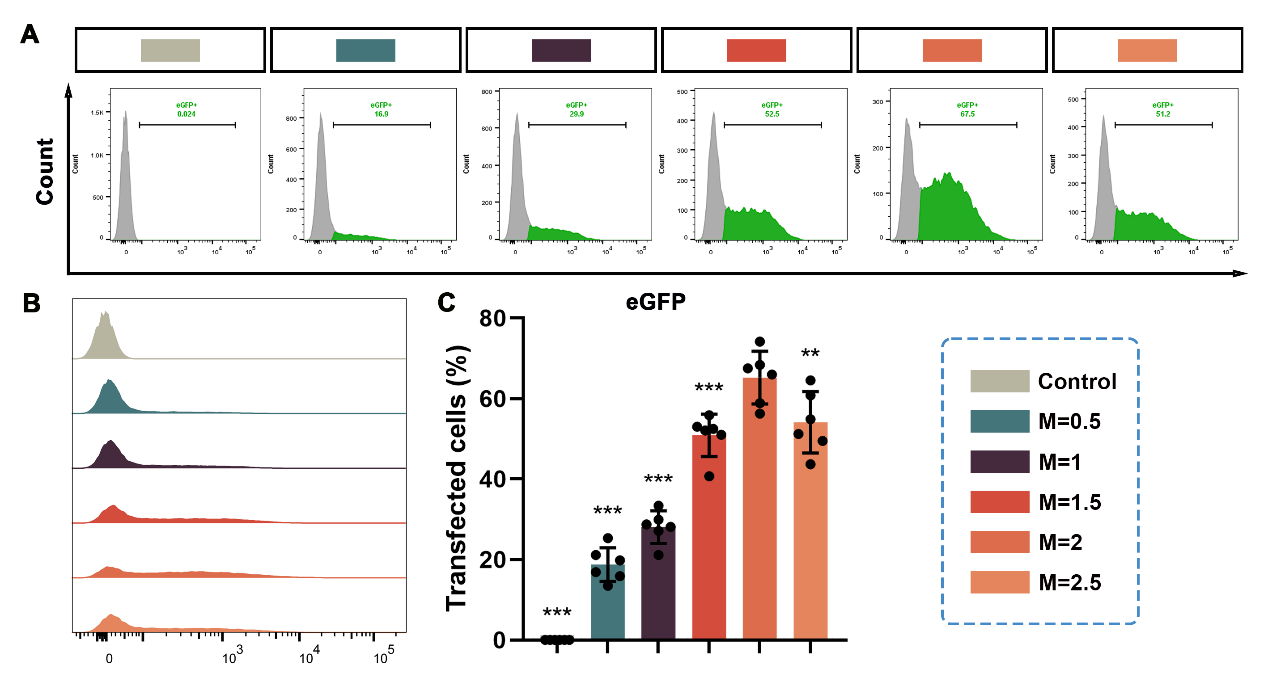


**Figure S7** (A, B) Transfection efficiency of pDNA@PEI-PBA polyplexes at different *M* ratios for rat fibroblast 208F cell lines was determined by flow cytometric (FCM) assays. (C) Transfection efficiency as the percentage of eGFP-positive cells was determined by FCM. (mean ± SD, *p < 0.05, **p < 0.01, ***p < 0.001, n = 6)

**
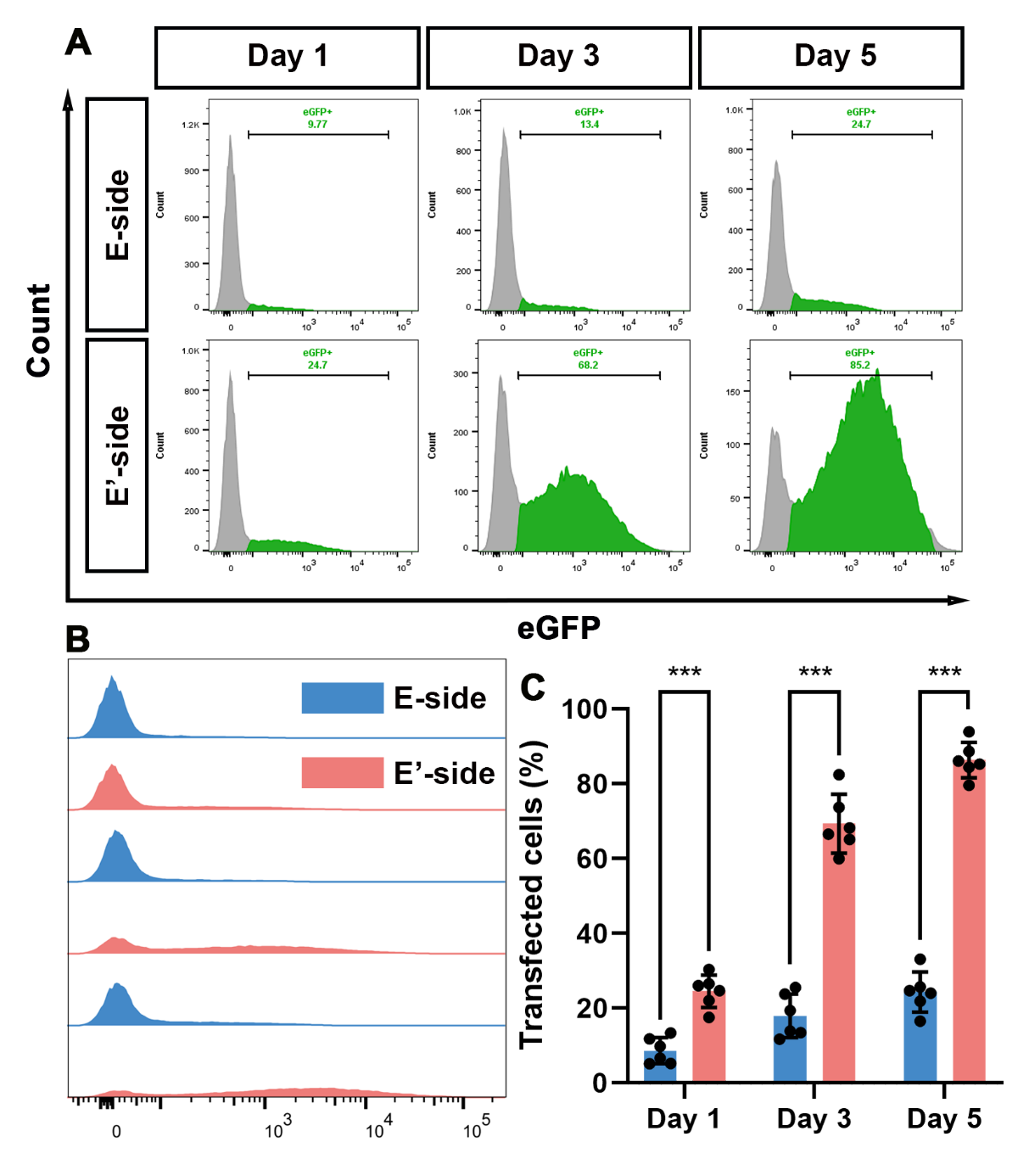
Figure S8** (A, B) FCM analysis of pDNA@PEI-PBA polyplexes-transfected 208F cells for 1, 3 and 5 days. (C) Transfection efficiency as the percentage of eGFP-positive cells was determined by FCM. (mean ± SD, *p < 0.05, **p < 0.01, ***p < 0.001, n = 6)

**
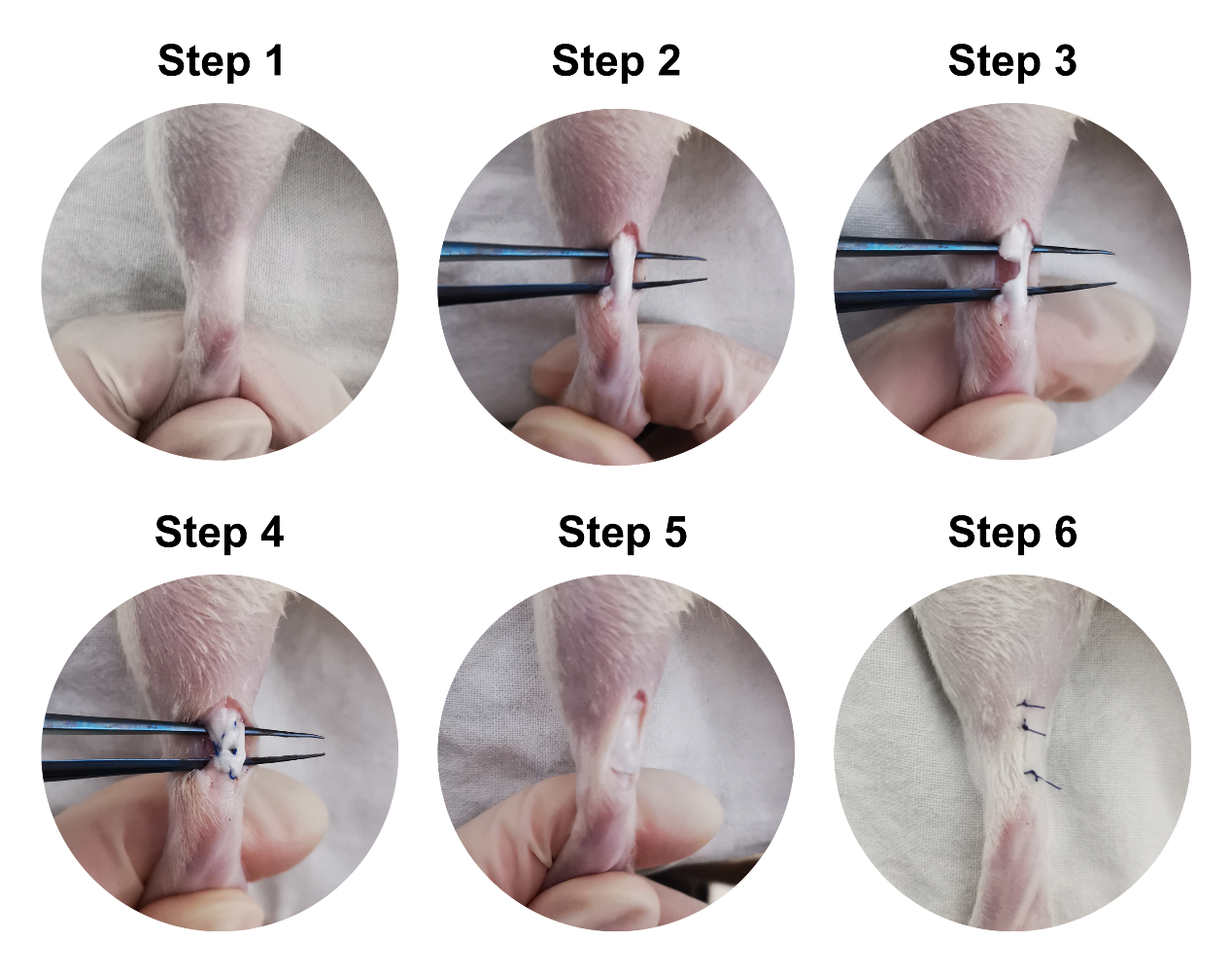
Figure S9** The surgical procedures of rat Achilles tendon injury model. Step 1: Shave and sterilize the surgical field. Step 2: Incise the skin and expose the Achilles tendon. Step 3: Transect the Achilles tendon in the middle. Step 4: Repair the ruptured tendon by means of modified Kessler technique. Step 5: Disinfect the anti-adhesion membrane and wrap the injured site with anti-adhesion membrane. Step 6: Close the skin incision and disinfect the skin with alcohol.

**
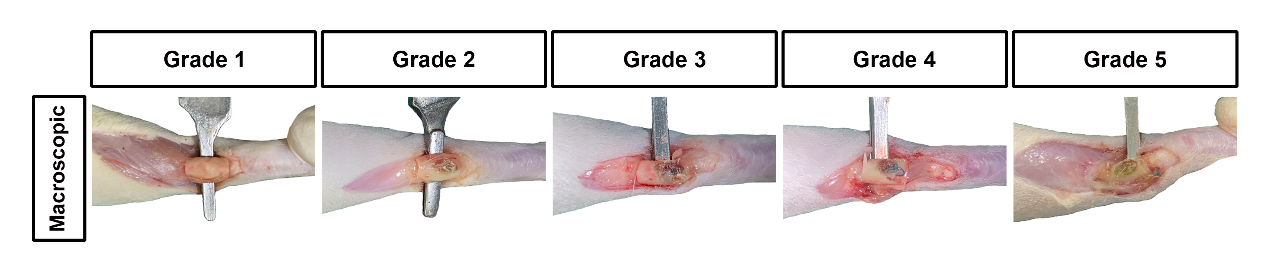
**

**Figure S10** The macroscopic evaluation standards for tendon adhesion. Tendon adhesion was assessed based on surgical observations at the repaired sites, using a grading scoring system from Grade 1-5: Grade 1, macroscopically no obvious adhesion tissues on the tendon surface; Grade 2, few adhesion tissues can be readily separated from tendon by blunt dissection; Grade 3, adhesion tissues covering less than 50% of the peritendinous area are separable with a blunt instrument; Grade 4, adhesion tissues covering 51-97.5% of the peritendinous area allow for separation with sharp dissection; Grade 5, adhesion tissues covering more than 97.5% of the peritendinous area can be separated by sharp dissection.

**
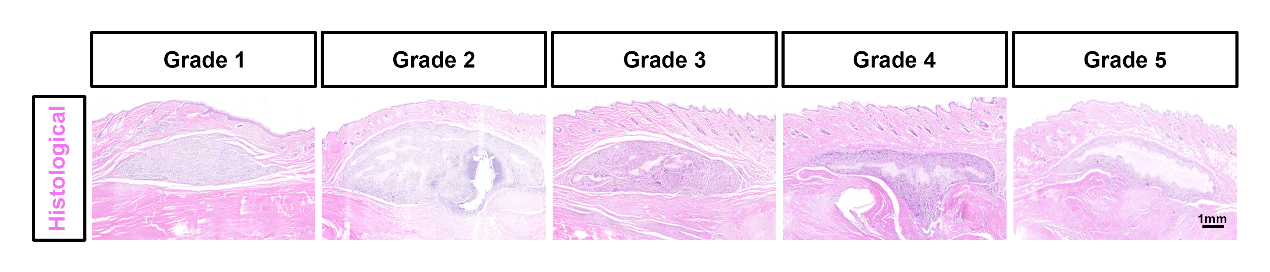
**

**Figure S11** The histological evaluation standards for tendon adhesion. A histological scoring system, based on the percentage of adhesion area at the repaired sites, was used to evaluate the extent of adhesion, ranging from Grade 1-5: Grade 1, no adhesion formation in the peritendinous area; Grade 2, less than 25% of peritendinous area occupied by adhesion tissues; Grade 3, 25-50% of peritendinous area occupied by adhesion tissues; Grade 4, 50-75% of peritendinous area occupied by adhesion tissues; Grade 5, more than 75% of peritendinous area occupied by adhesion tissues.

**
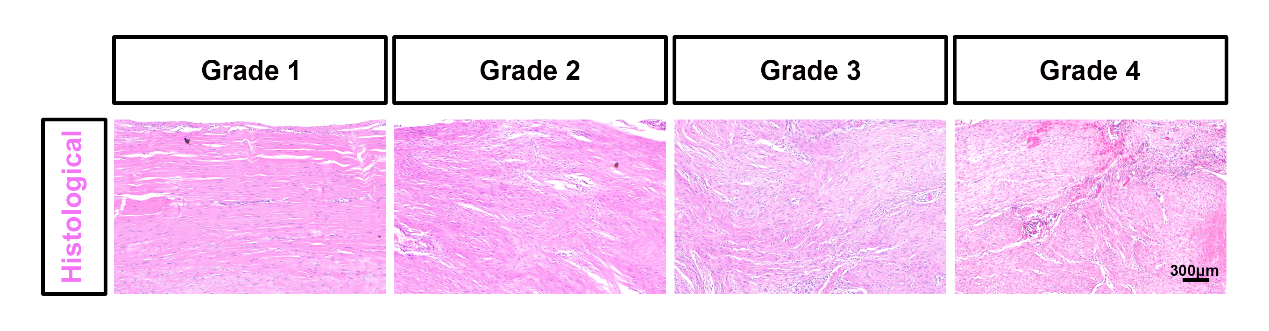
**

**Figure S12** The histological evaluation standards for tendon healing. Histological scoring of tendon healing was performed, with results categorized into Grades 1-4: Grade 1, excellent healing: regenerated tendon with well-organized collagen fiber arrangement and smooth surface without adhesion; Grade 2, good healing: good tendon continuity and rough surface with adhesion invasion; Grade 3, general healing: disordered collagen fiber arrangement with continuity interrupted by adhesion tissues; Grade 4, poor healing: repaired sites with failed healing or extensive adhesion tissue formation.

**
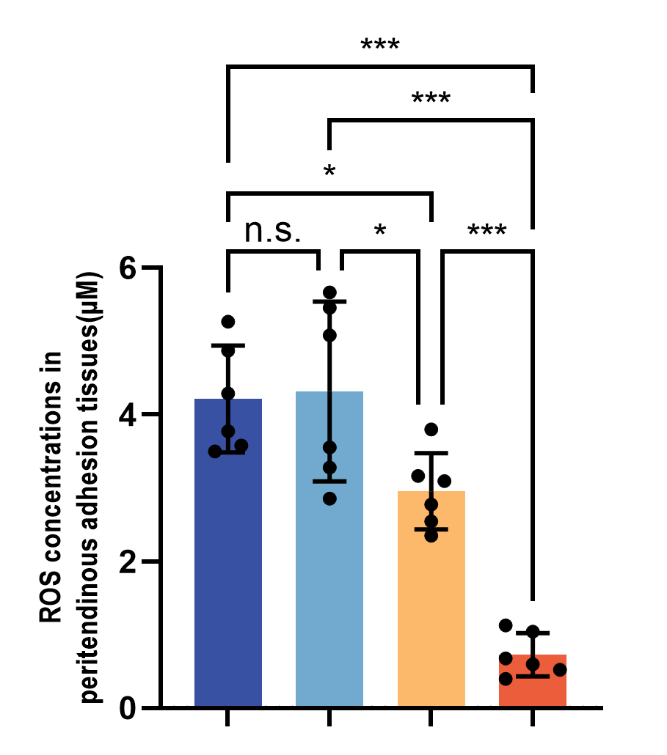
**

**Figure S13** ROS concentrations in peritendinous adhesion tissues from different groups. (mean ± SD, *p < 0.05, **p < 0.01, ***p < 0.001, n = 6)


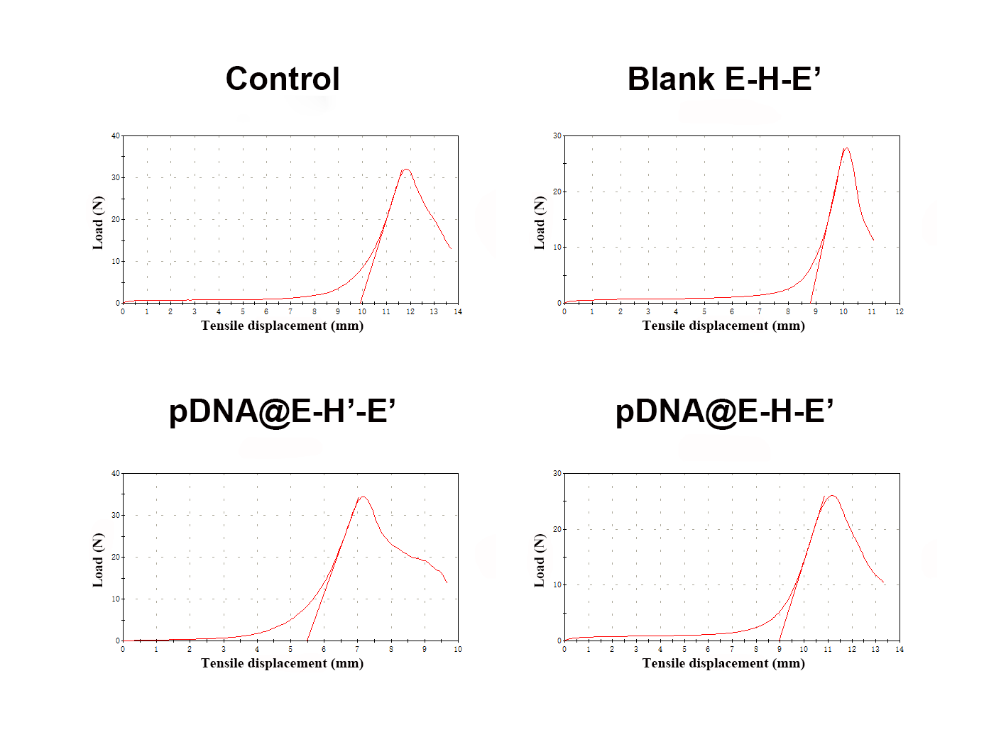


**Figure S14** Representative strength-tensile displacement curves among 4 groups were recorded by using a rheometer until the tendons were disrupted.


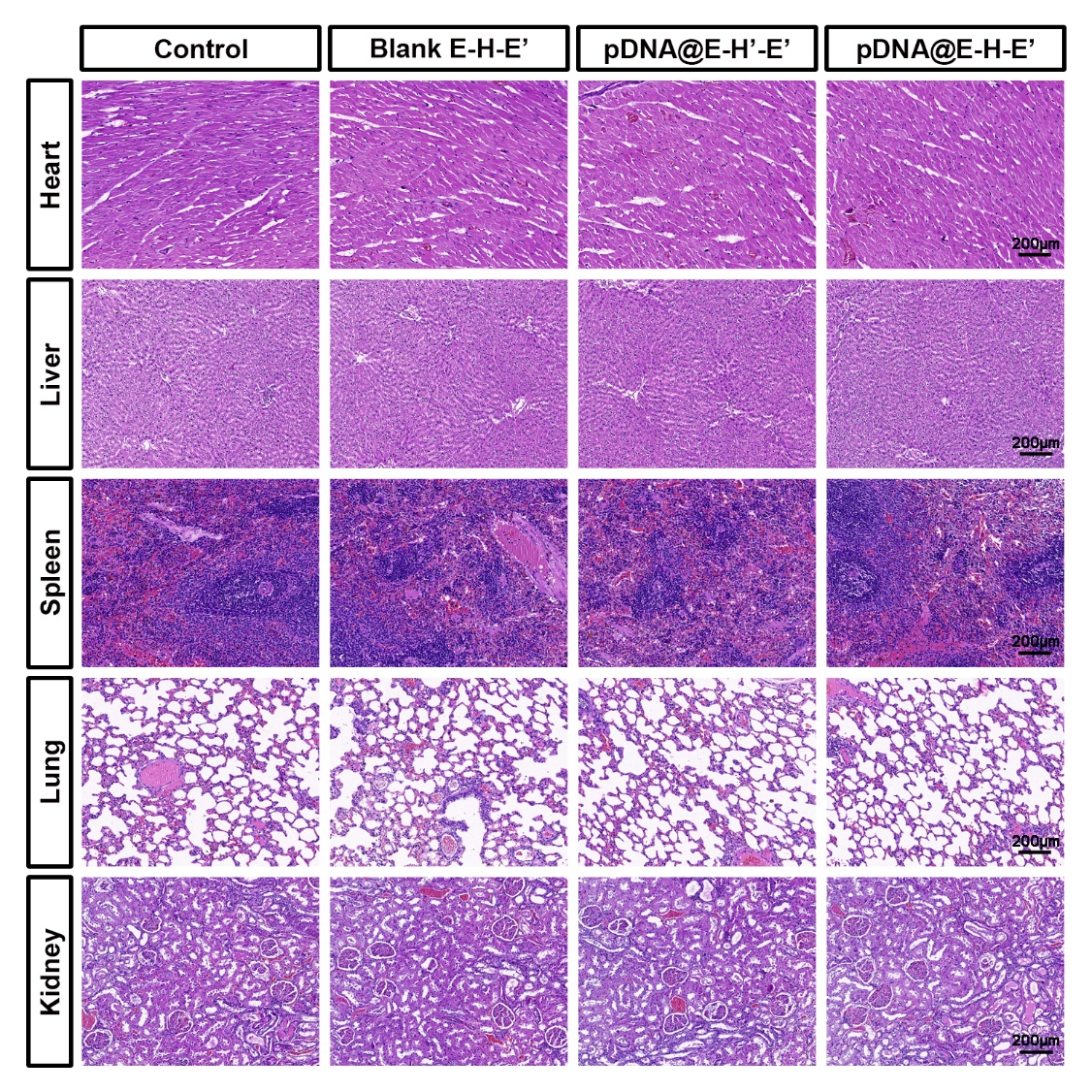
**Figure S15** Representative H&E staining images of rat heart, liver, spleen, lung and kidney from different groups.

**Table S1. Primer sequences used for rat fibroblast 208F cell lines**

| Gene | Primers (F=forward; R=reverse) |
| --- | --- |
| GAPDH | F: 5'-CCTGCACCACCAACTGCTTA-3'  R: 5'-GGCCATCCACAGTCTTCTGAG-3' |
| α-SMA | F: 5'-TTCGTGACTACTGCTGAGCG-3'  R: 5'-CTGTCAGCAATGCCTGGGTA-3' |
| Col Ⅰ | F: 5'-GGAGAGAGCATGACCGATGG-3'  R: 5'-GGTGGGAGGGAACCAGATTG-3' |
| Col III | F: 5'-CCTGAACTCAAGAGCGGAGAA-3'  R: 5'-CATGGCCTTGCGTGTTTGAT-3' |
|  |  |
